# Implementation and effectiveness of a school-based intervention to increase adherence to national school meal guidelines: a non-randomised controlled trial

**DOI:** 10.1017/S1368980023002938

**Published:** 2024-01-02

**Authors:** Jorunn Sofie Randby, Terje Ogden, Nanna Lien

**Affiliations:** 1 Department of Nutrition, Institute of Basic Medical Sciences, University of Oslo, Pb. 1046 Blindern, 0317 Oslo, Norway; 2 Department of Child and Adolescent Health, Norwegian Directorate of Health, Pb. 220 Skøyen, 0213 Oslo, Norway; 3 Norwegian Center for Child Behavioral Development, P.b. 7053 Majorstuen, 0306 Oslo, Norway

**Keywords:** Adherence, After-school service, Guidelines, Implementation, Norway, Primary schools, School meals

## Abstract

**Objective:**

Implementation of school meal guidelines is often inadequate, and evidence for effective implementation strategies for school-based nutrition interventions is limited. The aim of the present study was to examine the implementation and effectiveness of a multi-strategy implementation intervention to increase adherence to the Norwegian national school meal guideline.

**Design:**

The study was a school-based hybrid implementation effectiveness trial with a pre–post non-equivalent control group design, testing three implementation strategies: internal facilitation, training and an educational meeting.

**Setting:**

Primary schools and after-school services in two counties in south-east Norway.

**Participants:**

School principals, after-school leaders and class teachers from thirty-three schools in the intervention county and principals and after-school leaders from thirty-four schools in a comparison county.

**Results:**

There was a significant difference of 4 percentage points in change scores between the intervention and the comparison groups at follow-up, after adjusting for baseline adherence (*B* = 0·04, se *B* = 0·01, *t* = 3·10, *P* = 0·003). The intervention effect was not associated with the school’s socio-economic profile. School-level fidelity was the implementation dimension that was most strongly correlated (*r*_*s*_ = 0·48) with the change scores in the intervention group, indicating that principals’ support is important for gaining the largest intervention effects.

**Conclusions:**

A school-based intervention with low intensity, based on trained teachers as internal facilitators, can increase adherence to the national school meal guideline among Norwegian primary schools, irrespective of local socio-economic conditions. Implementation fidelity, at an organisational level, may be a useful predictor for intervention outcomes in schools.

School food policies may improve the school food environment, influence children’s dietary intake and impact on their long-term health^(1–4)^. Guidelines for school food provision most often address healthy menus and appropriate portion sizes or nutrient content^(5)^, but some also promote social aspects of school meals^(6–8)^. Implementation is, however, often inadequate^(9)^.

In Norway, advisory guidelines for school meals have existed since the 1970s, despite no universal food provision. Most schools rely on packed lunches and only offer subsidised subscription schemes for milk and fruit. However, the care service available to children in grades 1–4 both before and after school hours (‘the after-school service’) in most cases serve a meal in the afternoon, either warm or bread based^(10)^. In 2015, the national school meal guideline was substantially revised and disseminated in print to all schools. It comprises twenty-one recommendations relating to social and organisational aspects of mealtimes (time to eat, supervision, physical and social environment), nutritional quality of foods and drinks on offer, food safety and hygiene, as well as environmentally friendly practices^(6)^.

To promote guideline implementation, increased knowledge about effective implementation strategies is needed. To our knowledge, no trial to date has tested strategies to increase adherence to a comprehensive school meal guideline covering both nutrition, and social and organisational, aspects of school meals. Moreover, research on implementation variability is needed to better understand intervention mechanisms^(11–13)^.

## Definitions and research overview

Evidence shows that the quality of implementation matters^(14)^. *Implementation* may be defined as ‘a specified set of activities designed to put into practice an activity or program of known dimensions’^(15)^ (p. 5), implying that dissemination of practice guidelines, or training alone, is insufficient. The Active Implementation Drivers framework posits that developing competencies, making organisational changes and strengthening leadership are the most important drivers for implementation^(16)^. *Implementation strategies* have been defined as ‘methods or techniques used to enhance the adoption, implementation, and sustainability of a clinical program or practice’^(17)^ (p. 2). Various categorisations of implementation strategies exist, such as the Expert Recommendations for Implementing Change (ERIC) project, which identifies seventy-three discrete strategies^(18,19)^, and the Effective Practice and Organisation of Care (EPOC) taxonomy, which identifies twenty-two strategies^(20)^.

A review of implementation strategies showed that evidence of effective strategies in the field of school food policy is limited and of low quality^(9)^. Trial heterogeneity and inconsistent terminology complicated comparisons. All the twenty-seven trials used multiple strategies, but no two trials applied the same combination. Educational materials, educational outreach and educational meetings were the most common strategies. Nevertheless, of eighteen nutrition-related trials in the review, nine achieved significant effects for all or most implementation outcomes. Outcome measures comprised the percentage of programmes implemented, dichotomous measures and the number of completed activities over time. Only one trial used a nutrition practice change score as an outcome measure^(21)^, but that study did not achieve significant outcomes.

## Implementation barriers and enablers

The influence of contextual factors on the quality of implementation of school-based programmes is often overlooked^(22)^, despite being central in identifying the most promising implementation strategies. Knowledge of contextually relevant barriers and enablers for implementation of school food policies should therefore be identified along with synthesised knowledge of barriers and enablers for the specific setting (i.e. Norwegian primary schools). Two reviews of factors influencing school food policy implementation recently demonstrated a range of barriers and enablers^(23,24)^, some widely reported, others context-specific. The two reviews identified only one case study from Norway, for which the setting was secondary schools^(25)^. However, in a nation-wide, quantitative school meal survey in 2013, some barriers to guideline implementation in Norwegian primary schools were identified, including low guideline awareness among both principals and after-school leaders, unhealthy packed lunches and poor eating facilities. Low guideline adherence was evidenced by a high proportion of schools offering too little time to eat and a low proportion of after-school services offering fruit and vegetables daily^(10,26)^. Furthermore, low priority for social mealtimes has been identified among primary school teachers in a Norwegian study^(27)^. In a previous formative study in this project, identified barriers included weak administrative leadership linked to school meals, the lack of a school culture around meal practices, a noisy classroom climate undermining social meals and difficulties with teacher–parent collaboration concerning packed lunches^(28)^. Furthermore, after-school staff were largely unfamiliar with the guideline, had low formal competency in meal planning and food preparation, and operated without much influence or support from the school principal.

In the present study, the main objective was to test whether an intervention to provide schools with implementation support, based on strategies of internal facilitation, training and an educational meeting could increase the schools’ adherence to the national guideline on food and meals, in primary schools and after-school services. Second, we aimed to identify important implementation dimensions linked with increased adherence.

## Methods

### Study design and sample

We conducted a type II hybrid implementation effectiveness trial with a pre–post non-equivalent control group design (the ‘Food Ambassador study’)^(29,30)^. The study was conducted in collaboration with a public health project called ‘RØRE’ (‘MOVE’) in the county of Østfold. Østfold is a geographically small coastal county in south-east Norway, bordering Sweden. It is composed of eighteen municipalities and had a population of 300 000 in 2019, making it the sixth largest of Norway’s nineteen counties. Østfold had 35 000 children in obligatory schooling (grades 1–10) in 2019, across 133 schools. It ranks below the national average on most health and social indicators^(31)^. The MOVE project aimed to promote physical activity, healthy diets and good sleeping habits among schoolchildren of all ages. The project had been available to schools in Østfold since 2017, but exposure to diet-related activities had been limited to information about the national guideline and opportunities to apply for funding. In the collaboration for the Food Ambassador study, it was agreed that nutrition researchers would be responsible for designing and conducting the nutrition-related activities in the county of Østfold in the autumn 2019 (September–November). The MOVE project administration would be responsible for organising the physical meetings required to deliver the intervention. Buskerud county was identified as the best practice-as-usual comparison county, based on socio-economic^(31)^ and dietary indicators^(32)^, and being located at a similar distance to Oslo as Østfold. The comparison schools did not receive any intervention components.

Based on results in a previous study^(33)^, we calculated that about thirty schools were warranted in each group to detect a significant difference in adherence levels between the groups pre–post intervention. Each county had only about 100 eligible schools. Based on previous response rates in similar studies, we invited all eligible schools, both public and private, with primary schoolchildren aged 6–13 years (grades 1–7).

### Data collection

**Fig. 1:**
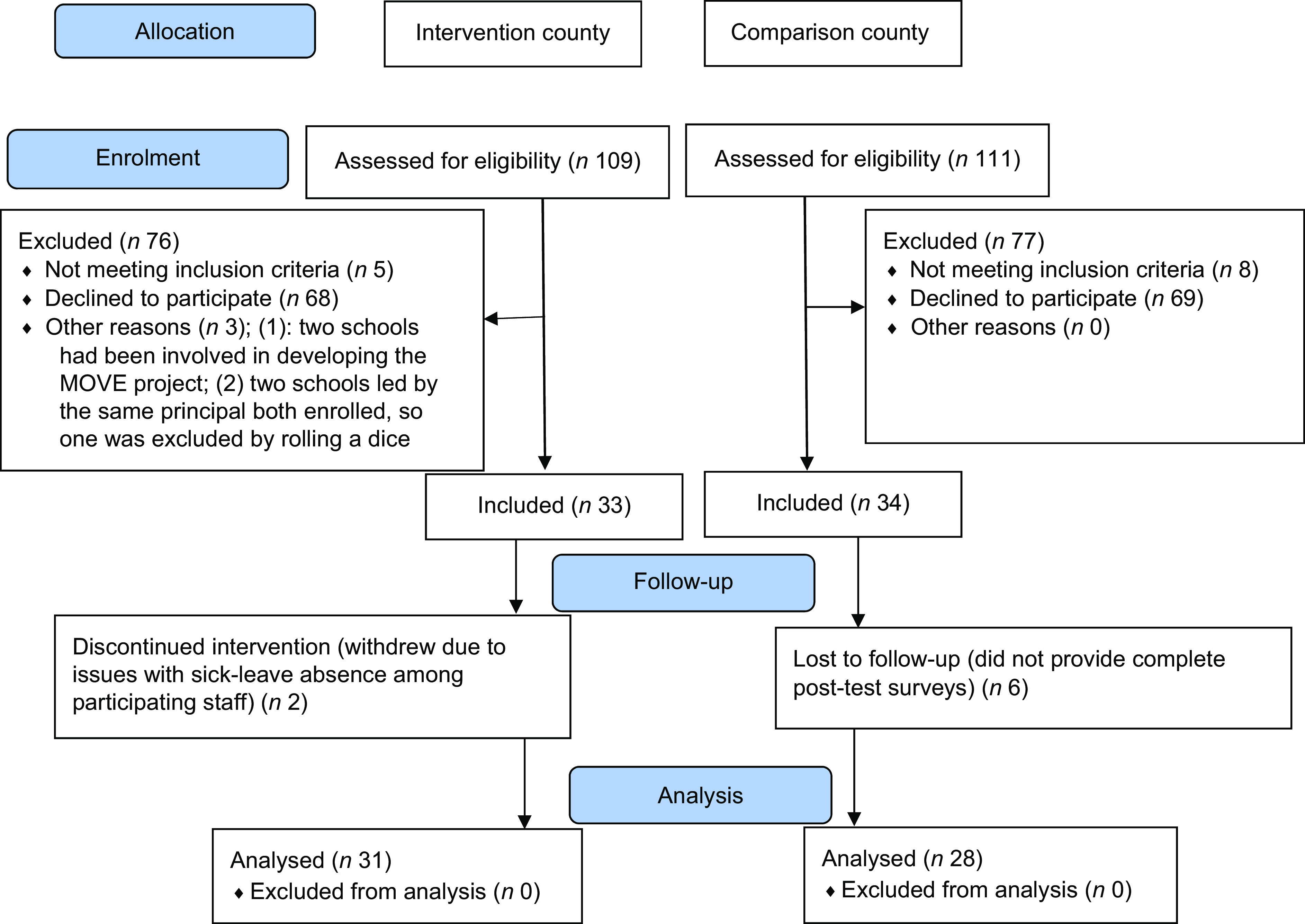
CONSORT flow chart describing study participation in the Food Ambassador study (*n* = number of schools)

Invitations were sent by email to school principals in April 2019. The inclusion criteria were the school comprising both lower (1–4) and higher (5–7) grades and offering an after-school service. In addition to thirteen schools that did not meet the inclusion criteria, two schools in the intervention county were excluded because they had helped develop MOVE (Fig. 1). Furthermore, as two schools with the same principal had enrolled, one was excluded (using throw of a dice) to ensure independence among participating schools. The baseline sample comprised thirty-three intervention schools and thirty-four comparison schools. Among the intervention schools in Østfold, eighteen had been engaged in the MOVE project for 1 year or more; however, there was no significant difference in baseline adherence scores between ‘MOVE schools’ and ‘non-MOVE schools’ participating in the Food Ambassador study (data not shown).

Baseline data on guideline adherence were collected in June 2019 and follow-up data in January/February 2020. Implementation data were collected throughout the intervention and through the follow-up questionnaires in the intervention group. Signed consent forms were collected from the principal, after-school leader and the appointed teacher who would receive training to be a ‘food ambassador’ in the intervention schools. In the comparison schools, principals and after-school leaders were informed via email that responding to the surveys implied consent to participate in the study as comparison schools.

Comparison schools that responded to the surveys at both time points received NOK 2000 as compensation. Intervention schools that were not part of MOVE received NOK 10 000 to cover for substitute teachers, and ‘MOVE schools’ received NOK 5000 (since some compensation was already given). The study protocol for compliance with data protection regulations was approved by the Norwegian Centre for Research Data on April 2, 2019 (ref. no. 457 729). The study was retrospectively registered as a trial in the ISRCTN registry on 7 August 2019 (ISRCTN12683953). A checklist for reporting implementation studies is available (Additional file 1).

### Implementation strategies

The Active Implementation Drivers framework^(34,35)^, recommending to target leadership drivers, organisational drivers and competency drivers, laid the foundation for the intervention development. Findings from a qualitative study on barriers and facilitators^(28)^ informed the selection of implementation strategies. Whole-school discussions on meal practices were identified as an important enabler for guideline implementation, leading us to select internal facilitation as the main implementation strategy. In addition, the content of the training sessions targeted some of the identified barriers, including a noisy classroom climate and difficulties with teacher–parent collaboration concerning packed lunches. Bearing in mind the possibility of potential future scale-up, the implementation of support measures was planned to be low cost and low intensity, relying to a large extent on local action being spurred by increased awareness and knowledge, networking effects and sharing of best practice among the schools. This is in line with current evidence supporting interventions that are simple, need limited resources and can be integrated into usual practice procedures^(9)^.

As shown in Table 1, three implementation strategies were employed to form the multi-strategy implementation intervention: internal facilitators as agents of practice change at their own school (teachers appointed as food ambassadors); educational meeting for all participants (principals, food ambassadors and after-school leaders); and training of food ambassadors and after-school leaders. These are consistent with three strategies (33, 15 and 19) in the ERIC project, all of which are rated as among the most promising strategies due to high feasibility and importance^(19)^. The external facilitators delivering the intervention were the first author (public health nutritionist), a senior adviser at the Norwegian Directorate of Health (clinical dietitian) and the two MOVE project leaders (one teacher and one public health nutritionist).

**Table 1:** Description of the Food Ambassador study’s three implementation strategies, according to recommendations^(17)^

	Educational meeting	Training	Internal facilitation
Actors	Principals, teachers, after-school leaders, external facilitators*	After-school leaders, teachers, external facilitators	Teachers (trained as ‘food ambassadors’)
Action	Information meeting led by the external facilitators, with info on the study, guideline content and rationale, the roles and responsibilities of the study participants, and time for questions and discussion	External facilitators provide one training session for after-school leaders covering dietary advice, children’s nutritional needs, meal planning, food procurement, sustainable choices, food safety and hygiene, helpful tools and other support material and initiatives, and workshop rationale and material. External facilitators provide two training sessions for the food ambassadors: first training session covers what constitutes favourable mealtime conditions and why it is important, dietary advice and children’s nutritional needs, the facilitation role, and implementation follow-up tasks; the second training session covers the sharing of experiences from the school-based workshops and a review of the school-based implementation plans, how to promote good collaboration with parents concerning packed lunches, and how to engage colleagues.	Food ambassadors conduct two workshops at own school: (1) with all class teachers and (2) with after-school staff. Each workshop starts with an introduction by the food ambassador (PowerPoint provided), followed by workshop participants filling out a one-page checklist for whether guideline recommendations are implemented (yes, no or partly). In plenary sessions, participants discuss which areas to focus on when going through the workshop guidance; this introduces each recommendation with a rationale and reflective questions for implementation. The workshop concludes with prioritised actions for improvement. The food ambassador sends the action plan to external facilitators before the second training session, informs the principal of the workshop outcomes and requests that time be set aside for follow-up discussions later in the semester, to review progress and re-commit to agreed action points
Target of the action	Knowledge about the guideline and their rationale, principal support, clarity about expectations	Detailed knowledge of the guideline recommendations and rationale, knowledge of children’s nutritional needs and how schools and after-school services can promote favourable mealtime conditions and healthy eating, and ensure food safety and hygiene	Ambassadors capable of acting as trustworthy change agents among colleagues, able to facilitate actionable discussions and hold the principal accountable for following meal practices in line with guideline recommendations
Temporality (all in 2019)	11 June	After-school leader training: 12th September Teacher trainings: (first) 3rd and 5th September; (second) 9th and 14th November	October
Dose	2 h	After-school leader training session: 3 h Ambassador training sessions: 3 h × 2	Ambassadors conduct two workshops at own school and after-school care
Implementation outcomes	Dose received; quality of delivery†	Dose received; quality of delivery; participant responsiveness	Dose received; quality of delivery; participant responsiveness; fidelity

* The external facilitators were the first author (public health nutritionist), a senior adviser at the Norwegian Directorate of Health (clinical dietitian) and two MOVE project leaders in the county administration of Østfold (one teacher and one public health nutritionist).

† These data could not be used because only half the participants put their school’s name on the evaluation form.

#### Internal facilitation

Harvey and Kitson’s Facilitation Guide^(36)^ served as the basis for shaping the role of the food ambassadors as local change agents. We particularly drew on the principles of reflective auditing processes when designing two school-based workshops that the food ambassadors would conduct, one with teacher colleagues and one with after-school staff. Input on the plans for the school-based workshops, given by staff at a non-participating school, substantially refined the workshop material. The material consisted of workshop guidance documents, modifiable PowerPoint presentations, implementation checklists and action plan templates. The prescribed schedule included an introduction by the food ambassador, individual review of practice through implementation checklists, a plenary discussion on focus areas guided by rationales and reflective questions for each guideline recommendation, and prioritised action points summarised in a school-based implementation plan. Finally, food ambassadors were to inform their principal of the action plan and to schedule a follow-up discussion later in the semester to review progress and re-commit to agreed actions.

#### Educational meeting

To promote principals’ buy-in to the intervention, the educational meeting in June was mandatory for principals, food ambassadors and after-school leaders. During the 2-hour face-to-face session, the external facilitators introduced the study, the guideline content and rationale, and the roles and responsibilities of the study participants, and also allowed time for questions and discussion.

#### Training

In the autumn, the external facilitators conducted separate training sessions for food ambassadors and after-school leaders, all of which were face-to-face and held during ordinary working hours. Two training sessions were organised for food ambassadors. For practical reasons these were held twice, attracting 15–17 participants each time. The first session (September) focused on the physical and social eating environment, nutritional needs of children and dietary advice, and the facilitation role and workshop method. The participating schools’ average adherence scores in various guideline areas at baseline were shown, and weak areas were highlighted. The second food ambassador training session (November) reviewed the schools’ implementation plans and workshop experiences. Two group discussions were organised, one about home-school collaboration concerning packed lunches and one about engaging colleagues. Food ambassadors were encouraged to work on formalising commitment to the guideline in the schools’ internal documents and to organise school-based activities.

One half-day training session for after-school leaders was organised in September, covering children’s nutritional needs, dietary advice, meal planning, food procurement, sustainable choices, food safety and hygiene, helpful tools, and other supporting material and initiatives. After-school leaders were introduced to the workshop rationale and material, although the food ambassador would lead the after-school workshop.

### Measures

#### Intervention outcomes

The effect of the implementation intervention was measured through two online questionnaires with acceptable test–retest reliability, designed to measure guideline adherence^(33)^. One questionnaire was designed for school principals, covering meal practices during school hours, and another for after-school leaders, covering meal practices during the after-school service. To keep the questionnaires short, the principal and after-school questionnaires pertain to twelve and fifteen of the twenty-one recommendations in the national guideline, respectively. Eight recommendations are covered by both questionnaires, whereas two are not measured by either because we were unable to operationalise them. As the after-school service is an integral part of the school and the principal has the overall responsibility for meals in both settings, the study was designed to target both settings equally. Adherence scores for meal practices in the two settings were merged to one overall adherence index for the whole school, comprising twenty-seven scores. An overview of questions and index scoring for each guideline domain is available as supplementary material (see Additional file 2).

#### Implementation outcomes

We measured implementation along four dimensions – quality of delivery, participant responsiveness, dosage and fidelity – primarily guided by the implementation quality approach of Meyers et al.^(37)^. Across the four dimensions, we assessed seventy-nine variables, using various data collection methods, including project records maintained by the principal investigator and questionnaires filled in by study participants (principals, after-school leaders and teachers). Each variable could yield 0, 0·5 (‘partly implemented’) or 1 point. Thus, an index score was calculated for each implementation dimension by dividing the points obtained by the maximum number of points available. Each dimension’s operationalisation, number of variables and data collection methods are summarised below. The questions, response options and scoring approach for all variables are available as supplementary material (see Additional file 3).

*Quality of delivery* refers to qualitative aspects of programme delivery, including inquiry into whether intervention delivery is perceived as responsive and sensitive to needs^(37)^. The construct carries resemblance to similar constructs in other implementation frameworks, such as the ‘acceptability’ and ‘appropriateness’ constructs in a paper on implementation outcomes by Proctor and colleagues^(38)^. The construct was measured by twenty-five variables collected through paper-based evaluation forms after the first training of the food ambassadors and the after-school leaders, as well as through two online surveys, answered by the food ambassador on completion of each workshop. The questions comprised participants’ assessments of session effectiveness (e.g. whether the presentations were clear and responsive and whether the meeting content was sensitive to needs) and implementer preparedness and enthusiasm. *Participant responsiveness* was measured through twenty-two variables collected through the online surveys after the workshops, the follow-up guideline adherence survey among principals and checklists of actions. Ten variables covered participation and involvement (e.g. the food ambassador’s response time for download of materials and ability to engage colleagues in discussions) and twelve variables covered engagement (e.g. food ambassadors’ use of suggested support material during the workshops and their completion of suggested project-related activities). *Dosage* was operationalised as ‘dose received’^(39)^ and measured through nine variables. The data were based on registered attendance of the study participants at the information meeting and trainings held by the external facilitators and the participation of teachers and other school staff during the school-based workshops, as reported by the food ambassadors. Finally, *fidelity* was conceptualised as integrity^(37)^ and measured through twenty-three variables collected through the online surveys after the workshops and additional questions in the follow-up adherence surveys. Building on previous research^(40)^, the *fidelity* dimension was divided into two aspects: programme integrity and practice/action integrity. Programme integrity deals primarily with decisions at an organisational level, such as ensuring necessary resources for implementation and support of the organisation’s leadership. Practice integrity, on the other hand, assesses how the individual practitioner utilises the method. Eleven variables covered the first aspect, labelled ‘school-level fidelity’ and twelve variables covered the second aspect, labelled ‘ambassador fidelity’. Table 2 provides an overview of the intervention and implementation outcomes measured in the study. The study’s logic model is available as supplementary material (see Additional file 4).

**Table 2:** Overview of guideline recommendations and implementation aspects providing the basis for evaluating outcomes in the Food Ambassador study

Guideline recommendations providing the basis for measuring the intervention outcome	
Meal organisation and social aspects	Meals should be arranged so as to be conducted at 3 to 4-hour intervals Physical arrangements should be made for meals that promote enjoyment of meals, socialisation, well-being and health Pupils should be given enough time to eat; at least 20 min Pupils should be supervised by an adult at mealtimes
Food and drink quality	Cold drinking water should be available at all times as a thirst quencher and to accompany meals Pupils should be offered schemes that ensure daily access to vegetables, fruit or berries Pupils should be offered schemes that ensure access to milk to accompany meals: reduced-fat semi-skimmed milk (0·7 % fat), semi-skimmed milk (1 % fat) and/or skimmed milk (0·1 % fat) Carbonated soft drinks, squash and other beverages containing added sugar or artificial sweeteners and caffeinated beverages should not be offered Bread and cereals in school meals should be high in fibre and wholegrains and low in fat, sugar and salt Bread toppings/spreads offered to pupils should be varied and always include fish and vegetables Any hot meals served should be a variety of fish, meat and vegetarian dishes Cooking oils and liquid and soft margarine should be used instead of hard margarine and butter Low-salt/Na foods should be given priority and the use of salt/Na as seasoning in food preparation and on meals should be limited Sugary and high-fat baked and other goods should be limited to special occasions Chocolate, confectionery, potato chips and other snacks should not be offered
Food safety and hygiene	Arrangements should be made to ensure hand-washing before meals Storage, preparation, serving and labelling of food must be carried out in compliance with rules and recommendations issued by the Norwegian Food Safety Authority Needs of pupils with food allergies or food intolerances should be accommodated
Sustainability	Eco-friendly practices should be aimed for to achieve minimal food waste and meal options in which plant-based foods and fish are focal

Implementation aspects providing the basis for measuring the implementation outcomes	
Quality of delivery	Participants’ assessments of session effectiveness and implementer preparedness and enthusiasm
Participant responsiveness	Participation and involvement Engagement
Dosage	Registered attendance
Fidelity	Programme integrity, labelled ‘school-level fidelity’ Practice integrity, labelled ‘ambassador fidelity’

### Data analysis

Statistical analyses were done using IBM SPSS Statistics 27. To compare school characteristics at baseline, χ^2^ tests (with Yate’s continuity correction) were conducted for all background variables. Fisher’s exact probability test was used whenever a cell had an expected count < 10.

A proxy for school-level socio-economic status (SES) was obtained from Statistics Norway’s open national database for school indicators^(41,42)^. SES data were available for thirty schools in each study group (due to data protection for very small schools). The proxy variable for SES indicates the school’s placement on a scale in a positive or negative direction from zero, which represents the national average. To categorise schools according to SES, the median (*M* = −0·80) of all schools with available SES data in the two counties (*n* 178) was used as a cut-off to classify participating schools as ‘low SES’ or ‘high SES’, similar to a previous study^(43)^. Binary logistic regression was used to test whether SES was a predictor for attrition.

Based on population categories used in national mapping surveys^(26)^ and reported frequencies, school locality was categorised as: ‘urban’ (≥ 20 000), ‘suburban’ (2000–20 000) or ‘rural’ (< 2000). School size was categorised as ‘small’ (< 100 pupils), ‘medium’ (100–299 pupils) and large (> 300 pupils), based on the national average of 220 pupils.

The study was powered to detect an effect size difference of 9 % between the groups, with 80 % power at a significance level of *P* ≤ 0·05. To assess the intervention outcome, two-sample Student’s *t* test was used to compare baseline adherence scores and evaluate unadjusted adherence change scores (pre–post adherence) between the groups. Standard multiple regression was used to compare the change scores between the groups, adjusted for baseline values, as recommended due to the possibility of regression to the mean. To avoid multi-collinearity, centred variables were used in the significance testing of an interaction effect between baseline adherence and groups on change scores. Sensitivity analysis was conducted by imputing baseline adherence scores for follow-up scores among drop-out schools (baseline observation carried forward).

Descriptive statistics were used to assess levels of implementation in the intervention group. To study associations between implementation processes and intervention outcomes, we used Pearson’s bivariate correlation analysis for normally distributed variables and Spearman’s rank-order correlations (*r*_*s*_) for non-normally distributed variables. Last, to check whether implementation variability could predict intervention outcomes, we conducted an exploratory multiple regression analysis with the implementation variables that correlated the highest with the intervention outcome as possible predictors.

## Results

### School and sample characteristics

The proportion of participating schools was 33 % in each county (see Fig. 1), representing two of three municipalities in both counties. The average school sizes of 285 (50–600) pupils in the intervention group and 293 (25–900) in the comparison group were similar, both slightly larger than their county averages of 265 and 247, respectively. There were nine combined schools (grades 1–10) in the intervention group (27 %) and five in the comparison group (15 %), compared with 21 % in both counties and 33 % nationally. Only two intervention schools (6 %) and one comparison school (3 %) were private, lower than the county proportions (8 % and 7 %, respectively) and the national average (9 %). The proportion of low-SES schools in the intervention county was significantly higher (60 %) than in the comparison county (36 %). However, the proportion of low-SES schools was similar between participating and non-participating schools in both the intervention (60 % and 58 %) and the comparison (40 % and 34 %) counties. At baseline, intervention schools were significantly less likely than the comparison schools to have principals who had 5 or more years of experience at the school and significantly more likely to have an after-school leader who was aware of the national guideline (Table 3). However, after attrition (see Fig. 1), these differences were no longer statistically significant.

**Table 3:** Baseline school characteristics in the Food Ambassador study†

	Total (*n* 67)		Intervention group (*n* 33)		Comparison group (*n* 34)		*P*-value‡
	*n*	%	*n*	%	*n*	%	
Setting (population)							
Urban	23	34·3	13	39·4	10	29·4	0·55
Suburban	28	41·8	14	42·4	14	41·2	1·00
Rural	16	23·9	6	18·2	10	29·4	0·39
School size							
Large	29	43·3	16	48·5	13	38·2	0·55
Medium	29	43·3	11	33·3	18	52·9	0·17
Small	9	13·4	6	18·2	3	8·8	0·31
School type							
Combined (grades 1–10)	14	20·9	9	27·3	5	14·7	0·24
Primary (grades 1–7)	53	79·1	24	72·7	29	85·3	0·24
School ownership							
Private schools	3	4·5	2	6·1	1	2·9	0·61
Public schools	64	95·5	31	93·9	33	97·1	0·61
SES profile§							
High SES	30	50·0	12	40·0	18	60·0	0·20
Low SES	30	50·0	18	60·0	12	40·0	0·20
Respondents of principal survey							
The respondent was the principal	52	77·6	25	75·8	27	79·4	0·78
Respondent has ≥ 5 years at the school	40	59·7	15	45·5	25	73·5	0·04*
Respondent aware of national guideline	51	76·1	25	75·8	26	76·5	1·00
Respondents of after-school survey							
The respondent was the after-school leader or person with assigned food service responsibility	61	91·0	31	93·9	30	88·2	0·67
Respondent has ≥ 5 years at the school	40	59·7	22	66·7	18	52·9	0·37
Respondent aware of national guideline	54	80·6	31	93·9	23	67·6	0·01*
Municipal initiatives							
Nutrition addressed at municipal level in the past 2 years	23	34·3	11	33·3	12	35·3	1·00

† The data source is the principal survey, except for the three items from the after-school leader survey and the SES variable.

‡ Significant results at *α* < 0·05 (two sided) are marked by an asterisk (*) in the table.

§ Data on school socio-economic status (SES) were not available for three intervention and four comparison schools in the baseline sample due to small school size. Thus, *n* 60 for these analyses, with *n* 30 in each group.

In the baseline school survey, fifty-two of the sixty-seven respondents were principals (78 %), twelve were chief educational officers (18 %), two were assistant principals (3 %) and one (1·5 %) was coordinator of the after-school service. For the after-school survey, sixty-one of the sixty-seven respondents were after-school leaders or the person assigned responsibility for the food service (91 %), four were other after-school employees (6 %) and two were principals (3 %). Across baseline and follow-up, seventy-seven people (of whom fourteen were male) responded to the principal questionnaire (due to five person changes in each study group) and seventy-four people (of whom nine were male) responded to the after-school questionnaire (due to five and two person changes in the intervention and the comparison groups, respectively). With the thirty-three food ambassadors (of whom five were male) in the intervention group, the total number of study participants was 184. In the intervention schools, some additional staff joined the training sessions and most of the teachers and after-school staff participated in the school-based workshops.

Among the eight schools that did not complete follow-up data collection, only four had principals as the respondent at baseline, whereas for the complete cases 81 % of the respondents were the school principal. There were no other considerable differences in measured school characteristics between the eight drop-out schools and the fifty-nine complete cases. SES was not a significant predictor of attrition (data available in Additional file 5).

There was no significant difference in baseline adherence scores between the intervention (mean = 0·66, sd = 0·09) and comparison group (mean = 0·68, sd = 0·08), *t* (57) = −1·04, *P* = 0·30 in the complete case analysis (*n* 59) (Table 4), nor was there a significant difference in the whole baseline sample (*n* 67) (see Additional file 5). SES was not correlated with baseline adherence (see Additional file 5).

**Table 4:** Adherence levels and change scores in the Food Ambassador study (*n* 59)

	Intervention group (*n* 31)		Comparison group (*n* 28)		Difference		*P*-value*
	Mean	sd/se	Mean	sd/se	Mean	se	
Baseline							
Mean adherence level	0·66	0·09	0·68	0·08	0·02	0·02	0·30
Follow-up							
Mean adherence level	0·74	0·05	0·71	0·07	0·03	0·02	0·07
	Mean	se	Mean	se	Mean	se	
Change score							
Unadjusted difference between baseline and follow-up	0·08	0·01	0·03	0·01	0·05	0·02	0·005

* Based on two-sample Student’s *t* test.

### Intervention effectiveness

There was a significant, unadjusted, mean difference at follow-up of 5 percentage points in change scores for adherence between the intervention (mean = 0·08, se = 0·01) and comparison (mean = 0·03, se = 0·01) groups, *t* (57) = 2·95, *P* = 0·005 (Table 4). An inverse relationship between change scores and baseline adherence was observed for both groups. This shows that schools with the lowest score at baseline increased their score the most, which is common in effectiveness studies due to their larger potential for change. A graphic illustration of this finding is available as supplementary material (see Additional file 6). During testing of assumptions for statistical analysis, this relationship was shown to constitute a significant interaction. After adjusting for baseline adherence scores and adding an interaction term to the regression model (Table 5), there was a 4 percentage point difference in change scores between the groups (*B* = 0·04, se *B* = 0·01, *t* = 3·10, *P* = 0·003), with *F*(3,55) = 25.16, *P* = 0·003. The significant difference between the groups remained in the sensitivity analysis (see Additional file 5). SES did not correlate with the change score (data not shown).

**Table 5:** Model for adjusted analysis of intervention effect in the Food Ambassador study (*n* 59)*

	*B*	The se for *B*	*t*	*P*-value†
1 (constant)	0·49	0·01	7·97	< 0·001
Intervention	0·04	0·01	3·10	0·003
Baseline adherence	−0·52	0·08	−6·71	< 0·001
Interaction (intervention × baseline adherence)	−0·37	0·16	−2·39	0·020

* Adjusted *R* ^2^ = 0·56.

† Based on standard multiple regression.

Several practice changes linked with the adherence increase in the intervention group were evident, of which the largest changes during school hours were more time to eat, improved availability of cold drinking water, reduced access to chocolate and snacks, better hand-washing practices, and better access to fruit and vegetables. For meal practices during the after-school service, the largest changes were reduced access to beverages with added sugar or sweeteners, improved food safety practices, variety of warm dishes of meat, fish, and vegetarian options, reduced access to chocolate and snacks, healthier bread toppings, and reduced access to sugary and high-fat baked goods. More details are available (see Additional file 7).

### Implementation outcomes

The mean implementation levels obtained by the intervention schools for each dimension or aspect are shown in Table 6, along with their correlation with the change score. High implementation levels were observed across most outcomes, with index scores of 41 % for engagement, 68 % for quality of delivery, 77 % for both participation and involvement and ambassador fidelity, 79 % for school-level fidelity and 80 % for dosage. The high score for dosage reflects the high participation rate during training; among the schools completing the study, only one missed the September ambassador training session, three missed the November one and two missed the training session for the after-school leaders.

**Table 6:** Implementation index scores and correlations with the change score among intervention schools in the Food Ambassador study (*n* 31)

Implementation dimensions (number of variables in the index score)	Implementation index score for each dimension				Correlation with change score for each dimension*
	Mean score	sd	95 % CI	Min–Max	Pearson’s (*r*)/Spearman’s ρ (*r*_*s*_) bivariate correlation
Quality of delivery (25)	0·68	0·15	0·63, 0·74	0·37–1·00	*r* = 0·20
Participant responsiveness					
Participation and involvement (10)	0·77	0·13	0·72, 0·81	0·40–0·95	*r*_*s*_ = 0·25
Engagement (12)	0·41	0·15	0·36, 0·47	0·08–0·75	*r* = −0·07
Dosage (9)	0·80	0·16	0·74, 0·86	0·19–1·00	*r* = −0·24
Fidelity					
School-level fidelity (11)	0·79	0·15	0·74, 0·84	0·45–1·00	*r*_*s*_ * =* 0·48
Ambassador fidelity (12)	0·77	0·15	0·72, 0·83	0·46–1·00	*r* = 0·21

* Tests of normality showed that two implementation aspects did not have normal distribution; therefore, Spearman’s ρ was used to assess correlation with the change score.

School-level fidelity was the implementation aspect most strongly correlated (*r*_*s*_ = 0·48) with the change score. We also tested whether any of the dimensions or aspects correlated with each other (see Additional file 5) and found a correlation between school-level fidelity and participation and involvement of *r*_*s*_ = 0·53. No other correlations were > 0·5, which is often used as cut-off for large correlations^(44)^.

An exploratory model testing whether school-level fidelity and participation and involvement predicted change scores among the intervention schools showed that only school-level fidelity was significant. Alone in the model, school-level fidelity explained 28 % of the variance in the change score, *F*(1,29) = 12.45, *P* = 0·001. The results indicate that school-level fidelity (*B* = 0·29, *P* = 0·001) is a good predictor of increased adherence.

## Discussion

The multi-strategy implementation intervention tested in the Food Ambassador study was associated with increased school adherence to the national school meal guideline. The effect was not associated with SES but was larger for schools with low, compared with high, baseline adherence. Furthermore, organisational support from the school principal was identified as a potentially important predictor for increased adherence.

Several of the practice changes effected by the intervention were unsurprising due to low baseline adherence in the particular guideline area and a strong focus during the intervention. However, a surprising finding was the negligible change related to accommodating for social meals. This was the guideline domain where the intervention schools scored the lowest at baseline and, therefore, received extra attention during the intervention. Previously identified barriers linked with promoting social and enjoyable meals may help to explain this, including a low priority given to school meals and a noisy classroom climate^(27,28)^.

The moderating effect of baseline adherence on intervention effectiveness may be linked to the varying degrees of effort required to make changes in different guideline areas and the fact that the low adhering schools had more areas to work with. This finding indicates, however, that schools with the lowest practice standards improved their practices the most, which is a positive finding.

It is difficult to compare the magnitude of the overall intervention effect with other studies in this field due to the heterogeneity of outcome assessments. In a review of implementation strategies^(9)^, only one nutrition-related trial used a continuous outcome measure, and this trial did not find a significant effect of the intervention^(21)^. However, our findings are in line with those of other multi-strategy implementation trials that have documented positive effects of increased guideline use. For example, Australian studies have shown improvements in children’s dietary intake in childcare centres^(45)^ and improvements in students’ purchases in school canteens^(46)^, and a Dutch study has shown improved availability of healthy products in the school cafeteria^(47)^.

Since the intervention effect was independent of the schools’ SES profiles, the effects of the intervention benefited children regardless of local SES conditions. This is consistent with findings in several Australian studies, including interventions to increase the implementation of fruit and vegetable breaks in schools^(43)^ and healthy school canteen policies^(46)^. Recent studies from Sweden have documented a larger impact of implementation of school food policies on children of low SES backgrounds^(4,48)^. However, still today few studies have evaluated the effects of school food interventions on different SES groups^(3,49)^.

In terms of discrete strategies, four out of the nine studies with effective implementation outcomes in the review of implementation strategies^(9)^ employed, among other things, a *local consensus process* ^(46,50–52)^, according to the terminology in the EPOC taxonomy^(20)^. We also used school-based consensus processes, but, as they depended on an internal facilitator whose role was broader than consensus building, we labelled our strategy *facilitation*. It is interesting that the EPOC taxonomy lacks *facilitation*, whereas the ERIC project presents *local consensus process* and *facilitation* as two separate strategies^(19)^. While recognising that delineation of strategies remains challenging, we maintain that *facilitation* describes our main implementation strategy better than *local consensus process* and believe that our study contributes to the evidence that trained internal facilitators may be a promising implementation strategy in school-based public health work.

The only implementation dimension with a low implementation score was engagement, which may be because the index included several items about encouraged, but not required, intervention activities. More surprising was the weak associations between all the implementation scores and the intervention outcome, with the exception of school-level fidelity, which may indicate poor validity of the implementation indices. However, it is possible that school-level fidelity is particularly important for changes to school meals. An implication of this finding is that distinguishing between organisational-level fidelity and practitioner-level fidelity may be valuable. However, it is pertinent to ask how closely related the school-level fidelity index is to implementation determinants such as administrative support and perceptions and attitudes among school staff, previously identified as important^(22)^.

### Study strengths and limitations

The use of implementation theory and frameworks, knowledge obtained in the project’s two formative studies and user involvement may have contributed to high levels of implementation and a low drop-out rate. Validated questionnaires for assessing the intervention outcomes increases confidence in the intervention effect estimate. Since responders to the intervention outcome surveys differed from the study’s main change agents, the risk of socially desirable responding may be reduced. Finally, we reduced the risk of selection bias by using comparable counties from which to recruit schools, the samples recruited from each county were representative of the county overall, and there were no considerable differences in measured background variables between the groups. Nevertheless, non-observed confounders cannot be ruled out and generalisations about the effect estimate must be done carefully.

Although the Norwegian school meal guideline is evidence-based, its effectiveness to improve children’s health and well-being has not yet been established. The effect estimate therefore refers only to increased guideline adherence. The assumed link between guideline use and outcomes in children is based on previous research documenting such links^(1–3,9,45,46)^. To a large extent, it operationalises the dietary recommendations. Using multiple methods and data sources does not make up for the lack of validated instruments for measuring the implementation outcomes. Programme adaptations are frequently made by teachers^(22)^ and therefore should be monitored and assessed^(37)^, preferably through post-intervention interviews^(53)^. This was, however, outside the scope of the present study. Finally, the small grants to participating schools may have influenced the selection, possibly reducing external validity.

## Conclusions

A relatively small implementation intervention based on internal facilitation, training and an educational meeting increased Norwegian primary schools’ adherence to the national school meal guideline. Active administrative leadership by the school principal was important for change. The present study may inform work on guideline development, implementation and monitoring in Norway and other jurisdictions, and it informs policymakers and practitioners of a promising intervention to increase the utilisation of normative tools for public health work in schools, irrespective of the school socio-economic profile.

## Supporting information

Randby et al. supplementary material 1 Randby et al. supplementary material

Randby et al. supplementary material 2 Randby et al. supplementary material

Randby et al. supplementary material 3 Randby et al. supplementary material

Randby et al. supplementary material 4 Randby et al. supplementary material

Randby et al. supplementary material 5 Randby et al. supplementary material

Randby et al. supplementary material 6 Randby et al. supplementary material

Randby et al. supplementary material 7 Randby et al. supplementary material

## Supplementary material

For supplementary material accompanying this paper visit https://doi.org/10.1017/S1368980023002938
