# Supplementary material for: Implementation and effectiveness of a school-based intervention to increase adherence to national school meal guidelines: a non-randomised controlled trial
Source: Public Health Nutr. 2024 Jan 2;27(1):e25. doi: 10.1017/S1368980023002938 (PMC10830359; doi:10.1017/S1368980023002938)
Supplement: Randby et al. supplementary material 2 — Randby et al. supplementary material [file S1368980023002938sup002.docx]

**Additional file 2: Scoring of the adherence index**

Part 1 – Adherence scores for meal practices during school hours

| **Guideline Recommendations** | **Variables in data file** | **Questions in the principal questionnaire** | **Scoring** | **Score number: calculated as** |
| --- | --- | --- | --- | --- |
| Meals should be arranged so as to be conducted at 3- to 4-hourly intervals | b11/p7 | Which meals/breaks are organized daily for the pupils?   - Breakfast (school/after-school service) - Lunch break before 10:30 - Lunch break between 10:30 and 12:00 - Lunch break after 12:00 - Fruit/vegetable break - Meal in the afternoon (school/after-school service) - Other meals - Don’t know | Lunch break between 10:30 and 12:00 = 1  Not lunch break between 10:30 and 12:00 = 0 | Score 1:  0, 1 or 2 points divided by 2 |
|  | b13/p9 | Does the school offer simple foods (e.g. crispbreads) in cases where pupils do not have a packed lunch? | Yes, pupils are offered food = 1  No, teacher finds a solution through sharing others’/own food in the classroom; No, students find their own solutions; Don’t know = 0 |  |
| Physical arrangements should be made for meals that promote enjoyment of meals, socialization, well-being and health | b27/p23 | Where do the pupils normally eat lunch (1st to 4th grades)? | In the classroom; In the canteen; Other place = 1  In the corridor; Don’t know = 0 | Score 2:  0–7 points divided by 7 |
|  | b28/p24 | Where do the pupils normally eat lunch (5th to 7th grades)? | In the classroom; In the canteen; Other place = 1  In the corridor; Don’t know = 0 |  |
|  | b30/p26 | How often does conversation (no organized activity) constitute most of the lunch break for 1st to 4th grades? | Every day; 3 or 4 days/week = 1  1 or 2 days/week; 1–3 days/month; Don’t know = 0 |  |
|  | b31/p27 | How often does a TV/screen/smartboard constitute most of the lunch break for 1st to 4th grades? | 1–3 days/month or less = 1  Every day; 3 or 4 days/week; 1 or 2 days/week; Don’t know = 0 |  |
|  | b33/p29 | How often does conversation (no organized activity) constitute most of the lunch break for 5th to 7th grades? | Every day; 3 or 4 days/week = 1  1-2 days/week; 1–3 days/month; Don’t know = 0 |  |
|  | b34/p30 | How often does a TV/screen/smartboard constitute most of the lunch break for 5th to 7th grades? | 1–3 days/month or less = 1  Every day; 3 or 4 days/week; 1 or 2 days/week; Don’t know = 0 |  |
|  | b39/p35 | How does the school define the time used for being present while the pupils eat lunch? | Supervision/inspection time = 1  Teaching time; Partly supervision and partly teaching; Don’t know **=** 0 |  |
| Pupils should be given enough time to eat − at least 20 minutes | b36/p32 | How much time do the pupils have available for the act of eating in 1st to 4th grades? | 20–24 min; 25–29 min; ≥30 min **=** 1  <10 min; 10–14 min; 15–19 min; **=** 0 | Score 3:  0, 1 or 2 points divided by 2 |
|  | b38/p34 | How much time do the pupils have available for the act of eating in 5th to 7th grades? | 20–24 min; 25–29 min; ≥30 min **=** 1  <10 min; 10–14 min; 15–19 min; **=** 0 |  |
| Pupils should be supervised by an adult at mealtimes | b41/p37 | During how much of the lunch break is an adult present together with the pupils (1st to 4th grades)? | All of it **=** 1  Parts of it; No adult is present during the lunch break; Don’t know **=** 0 | Score 4:  0, 1 or 2 points divided by 2 |
|  | b42/p38 | During how much of the lunch break is an adult present together with the pupils (5th to 7th grades)? | All of it **=** 1  Parts of it; no adult is present during the lunch break; don’t know **=** 0 |  |
| Cold drinking water should be available at all times as a thirst quencher and to accompany meals | b20/p16 | Do the pupils have access to drinking water in the following ways?   - Water from the tap in classrooms/dining rooms - Water dispenser - Water fountain - Water jugs (in canteen/classroom/dining room) - Don’t know | Yes, in one way or more **=** 1  No to all; Don’t know = 0 | Score 5:  0, 1 or 2 points divided by 2 |
|  | b21/p17 | Does the school have a common routine for students’ access to drinking water during class? | Yes, common routine allowing pupils to drink; Yes, common routine that pupils must wait until recess = 1  No; Don’t know **=** 0 |  |
| Pupils should be offered schemes that ensure daily access to vegetables, fruit or berries |  | Do the pupils have access to fruit/vegetables/berries at school in any of the following ways?   - No access - Yes, free of cost to all pupils - Yes, subscription scheme paid by parents - Yes, fruit/vegetables may be bought in canteen/sales point - Yes, in the after-school service - Yes, through a different scheme - Don’t know | No access; Don’t know **=** 0  If pupils have access every = 1 (see follow-up questions)  Every day = 1  3 or 4 days/week; 1-2 days/week; 1–3 days/month = 0 | Score 6:  0 or 1 point |
|  | b16/p12 | How often are free fruit/vegetables available? |  |  |
|  | b17/p13 | How often is the subscription scheme available? |  |  |
|  | b18/p14 | How often are fruit/vegetables available in canteen/sales point? |  |  |
|  | b19/p15 | How often are fruit/vegetables available through a different scheme? |  |  |
| Pupils should be offered schemes that ensure access to milk to accompany meals: reduced-fat semi-skimmed milk (0.7% fat), semi-skimmed milk (1% fat) and/or skimmed milk (0.1% fat) | b24/p20 | How often is milk available? | Every day **=** 1  3 or 4 days/week; 1 or 2 days/week; 1–3 days/month; Don’t know **=** 0 | Score 7:  0, 1 or 2 points divided by 2 |
|  | b25/p21 | Are the pupils usually offered the following types of milk?   - Whole milk (3.9–4.1% fat, red) - Semi-skimmed milk (1.0–1.2% fat, dark pink) - Semi-skimmed milk (0.5–0.7% fat, green) - Skimmed milk (0.1% fat, light pink) - Flavoured milk (cocoa/raspberry) - Lactose-free/lactose-reduced milk - Juice (apple/orange) - Vegetable drinks of soy/oat/almond/rice - Other - Don’t know | Whole milk **=** 0 (if this is offered, 0 is given irrespective of the other available milk options)  Any other milk option = 1 |  |
| Arrangements should be made to ensure hand-washing before meals | b43/p39 | Roughly how many pupils wash their hands with soap and water before eating in 1st to 4th grades? | Nearly all **=** 1  More than half; About half; Less than half; Almost none; Don’t know **=** 0 | Score 8:  0–5 points divided by 5 |
|  | b45/p41 | Roughly how many pupils wash their hands with soap and water before eating in 5th to 7th grades? | Nearly all **=** 1  More than half; About half; Less than half; Almost none; Don’t know **=** 0 |  |
|  | b44/p40 | Does an adult monitor whether pupils wash their hands in 1st to 4th grades? | Yes **=** 1  No; Don’t know **=** 0 |  |
|  | b46/p42 | Does an adult monitor whether pupils wash their hands in 5th to 7th grades? | Yes **=** 1  No; Don’t know **=** 0 |  |
|  | b47/p43 | Does the school have routines for hand hygiene before pupils eat, when on excursions without access to water and soap? | Yes, school/teacher brings disinfectant **=** 1  No, pupils eat without washing hands; No, but bringing disinfectant is encouraged; Don’t know **=** 0 |  |
| The storage, preparation, serving and labelling of food must be carried out in compliance with rules and recommendations issued by the Norwegian Food Safety Authority | b55/p51 | Is the responsibility for controlling that the fridge/cold room remains at the recommended temperature assigned to an adult? | Yes **=** 1  No; Don’t know **=** 0  Not relevant is not counted | Score 9:  0, 1 or 2 points divided by 2 |
|  | b56/p52 | Is the school/after-school service’s food and drink availability, or food handling, reported to the Food Safety Authority? | Yes **=** 1  No; don’t know **=** 0  Not relevant is not counted |  |
| Carbonated soft drinks, squash and other beverages containing added sugar or artificial sweeteners and caffeinated beverages should not be offered | b54/p50 | In the course of the year, how often may pupils drink carbonated soft drinks, squash or other beverages containing added sugar or artificial sweeteners during school hours? | Never; 1 or 2 times/year = 1  Once a week or more often; 1–3 times/month; 5–9 times/year; 3 or 4 times/year; Don’t know = 0 | Score 10:  0 or 1 point |
| Sugary and high-fat baked and other goods should be limited to special occasions | b50/p46 | Are birthdays celebrated with cake/ice-cream/sweet buns, etc. during school hours? | No, birthdays are celebrated in ways other than with food; No, birthdays are not celebrated in school = 1  Yes, separately for each pupil; Yes, a common celebration weekly; Yes, a common celebration monthly, Don’t know = 0 | Score 11:  0–3 points divided by 3 |
|  | b51/p47 | Which of the following foods are used occasionally to reward pupils/classes for good work or behavior?   - We don’t use food as rewards - Chocolate/candy/potato chips, etc. - Ice-cream/cookies/cake/sweet buns, etc. - Fruit/berries, etc. - Hot dogs/pizza, etc. | We don’t use food as rewards; Fruit/berries, etc. **=** 1  Chocolate/candy/potato chips, etc.; Ice-cream/cookies/cake/sweet buns, etc.; Hot dogs/pizza, etc. = 0 |  |
|  | b52/p48 | In the course of the year, how often may pupils eat cake, ice-cream, sweet buns, cookies, etc. during school hours? | Never; 1 or 2 times/year; 3 or 4 times/year **=** 1  Once a week or more often; 1–3 times/month; 5–9 times/year; Don’t know **=** 0 |  |
| Chocolate, confectionery, potato chips and other snacks should not be offered | b53/p49 | In the course of the year, how often may pupils eat chocolate, candy, potato chips, etc. during school hours? | Never; 1 or 2 times/year **=** 1  Once a week or more often; 1–3 times/month; 5–9 times/year; 3 or 4 times/year; Don’t know **=** 0 | Score 12:  0 or 1 point |
| Score for school hours |  |  |  | Maximally 12 points |

Part 2 – Adherence scores for meal practices during after-school service

| **Guideline Recommendations** | **Variables in data file** | **Questions in the after-school care questionnaire** | **Scoring** | **Score number:**  **calculated as** |
| --- | --- | --- | --- | --- |
| Meals should be arranged so as to be conducted at 3 to 4-hour intervals | sb13/sp10 | How, and how often, are meals organized after school hours, in the after-school service (food brought from home)? | Summarize the three variables to see if the service organizes a minimum of 5 meals per week. If so, 1 point is given; otherwise zero. | Score 1:  0 or 1 point |
|  | sb14/sp11 | How, and how often, are meals organized after school hours, in the after-school service (served bread-based meal)? |  |  |
|  | sb15/sp12 | How, and how often, are meals organized after school hours, in the after-school service (served hot meal)? |  |  |
| Cold drinking water should be available at all times as a thirst quencher and to accompany meals | sb41/sp37 | Do the pupils have access to drinking water in the following ways?   - Water from the tap in classrooms/dining rooms - Water dispenser - Water fountain - Water jugs - Don’t’ know | Yes, in one way or more **=** 1  No to all; Don’t know = 0 | Score 2:  0 or 1 point |
| Pupils should be offered schemes that ensure daily access to vegetables, fruit or berries | sb17/sp13 | Is a separate break for consumption of fruit/vegetables/berries organized in the after-school service 1 day per week or more often? | Yes, and products are served: see follow-up question (sb18/sp14) for points  Yes, and products are brought from home; No; Don’t know **=** 0 | Score 3:  0–2 points divided by 2 |
|  | sb18/sp14 | How often are fruit/vegetables/berries served in a separate break in the after-school service? | 5 days/week = 1  1–3 days/month; 1 day/week; 2 days/week; 3 days/week; 4 days/week; Don’t know = 0 |  |
|  | sb30/sp26 | For how many of the bread/crispbread meals are fruit/berries served as a side dish or spread? | All; Almost all = 1  More than half; About half; Less than half; Almost none; None; Don’t know = 0 |  |
| Pupils should be offered schemes that ensure access to milk to accompany meals: reduced-fat semi-skimmed milk (0.7% fat), semi-skimmed milk (1% fat) and/or skimmed milk (0.1% fat) | sb35/sp31 | For how many of the bread/crisp bread meals in after-school service is milk available? | All; Almost all = 1  More than half; About half; Less than half; Almost none; None; Don’t know = 0 | Score 4:  0–3 points divided by 3 |
|  | sb36/sp32 | Are the pupils usually offered the following types of milk?   - Whole milk (3.9–4.1% fat, red) - Semi-skimmed milk (1.0–1.2% fat, dark pink) - Semi-skimmed milk (0.5–0.7% fat, green) - Skimmed milk (0.1% fat, light pink) - Lactose-free/lactose-reduced milk - Vegetable drinks of soy/oat/almond/rice - Don’t know | Whole milk **=** 0 (if this is offered, 0 is given irrespective of the other available milk options)  Any other option alone or in combination = 1 |  |
|  | Sb37/sp33 | Which of the following milk-based drinks are offered 1 day per week or more often?   - Chocolate milk - Milk with chocolate powder added - Hot chocolate - None of the above - Don’t know | None of the above = 1  Any other option alone or in combination = 0 |  |
| The storage, preparation, serving and labelling of food must be carried out in compliance with rules and recommendations issued by the Norwegian Food Safety Authority | Sb53/sp49 | Where do employees wash their hands before handling/preparing food? | Separate sink for handwashing in the kitchen = 1  Bathroom sink; Kitchen sink used for handwashing and food handling; Don’t know = 0 | Score 5:  0–4 points divided by 4 |
|  | Sb54/sp50 | Do new employees get a briefing on routines for food safety? | Yes, they get a walk-through of routines = 1  No; No, but they are asked to familiarize themselves with the rules; Don’t know = 0 |  |
|  | Sb55/sp51 | Is the food handling/food service reported to the Food Safety Authority*?* | Yes = 1  No; Don’t know = 0  Not relevant is not counted |  |
|  | Sb56/sp52 | Do you have a written protocol for self-monitoring, listing the requirements in the food safety regulation that are relevant to after-school services? | Yes = 1  No; Don’t know = 0  Not relevant is not counted |  |
| The needs of pupils with food allergies or food intolerances should be accommodated | Sb47/sp43 | Is the food service customized to pupils with food allergy and food intolerance? | Yes, the service ensures that these pupils receive equally good alternatives = 1  No, pupils with food allergy/intolerances must bring their own food; Don’t know = 0  Not relevant is not counted | Score 6:  0 or 1 point |
| Carbonated soft drinks, squash and other beverages containing added sugar or artificial sweeteners and caffeinated beverages should not be offered | Sb52/sp48 | Which of the following sugar-containing beverages are available 1 day per week or more often?   - Squash - Iced tea - Carbonated soft drinks - Other beverages containing sugar or artificial sweeteners - None of these types - Don’t know | None of these types = 1  Any other option alone or in combination = 0 | Score 7:  0 or 1 point |
| Bread and cereals in school meals should be high in fibre and whole grains and low in fat, sugar and salt | Sb20/sp16 | Which of the following cereals are available in the after-school service once a week or more often? | Breakfast cereals; Oats/oatmeal; None of these options **=** 1  Cornflakes; Sweetened cereals; Don’t know **=** 0  Not relevant is not counted | Score 8:  0–3 points divided by 3 |
|  | Sb22/sp18 | What type of bread is served? | Just whole-grain; More whole grain than white = 1  As much whole-grain as white; Less whole-grain than white; Just white; Don’t know = 0  Not relevant is not counted |  |
|  | Sb24/sp20 | What type of crispbread is served? | Just whole-grain; More whole grain than white = 1  As much whole-grain as white; Less whole-grain than white; Just white; Don’t know = 0  Not relevant is not counted |  |
| Bread toppings/spreads offered to pupils should be varied and always include fish and vegetables | Sb25/sp21 | How many different spreads are available during the bread-based meals? | 3 or 4; 5 or 6; 7 or more = 1  1 or 2 = 0  We don’t serve bread is not counted | Score 9:  0–6 points divided by 6 |
|  | Sb26/sp22 | To what degree is the Keyhole^a^ used when purchasing for selection of healthier spread options? | To a large degree; To a fairly large degree = 1  To neither a large nor a small degree; To a fairly low degree; To a low degree; Don’t know = 0  Not relevant is not counted |  |
|  | Sb28/sp24 | During how many of the bread-based meals are fish spreads served? | All = 1  Almost all; More than half; About half; Less than half; Almost none; None; Don’t know = 0  Not relevant is not counted |  |
|  | Sb29/sp25 | During how many of the bread-based meals are vegetables served as a side dish or spread? | All = 1  Almost all; More than half; About half; Less than half; Almost none; None; Don’t know = 0  Not relevant is not counted |  |
|  | Sb31/sp27 | During how many of the bread-based meals is jam served? | None = 1  All; Almost all; More than half; About half; Less than half; Almost none; Don’t know = 0  Not relevant is not counted |  |
|  | Sb32/sp28 | During how many of the bread-based meals is chocolate spread served? | None = 1  All; Almost all; More than half; About half; Less than half; Almost none; Don’t know = 0  Not relevant is not counted |  |
| Any hot meals served should be a variety of fish, meat and vegetarian dishes | Sb43/sp39 | Is fish served every fifth hot meal or more often? | Yes **=** 1  No; Don’t know = 0  Not relevant is not counted | Score 10:  0–2 points divided by 2 |
|  | Sb44/sp40 | Is a vegetarian dish (as main) served every fifth hot meal or more often? | Yes **=** 1  No; Don’t know = 0  Not relevant is not counted |  |
| Cooking oils and liquid and soft margarine should be used instead of hard margarine and butter | Sb33/sp29 | Which of the following types of oils/butter/margarine is usually available to put on bread/crisp bread? | Soft margarine (Brelett, Soft Flora, Vita Hjertego, etc.) **=** 1  Bremykt (semi-soft butter/oil mix); Hard margarine/butter (real dairy butter, Melange, etc.) = 0 | Score 11:  0–2 points divided by 2 |
|  | Sb45/sp41 | Which of the following types of butter/margarine is usually used in cooking? | Oil (rapeseed, sunflower, soy, etc.); Liquid margarine (liquid Bremykt, Melange, etc.); Soft margarine (Brelett, Soft Flora, Vita Hjertego, etc.) = 1  Bremykt (semi-soft butter/oil mix); Hard margarine/butter (real dairy butter, etc.) = 0 |  |
| Low-salt/sodium foods should be given priority and the use of salt/sodium as seasoning in food preparation and on meals should be limited | Sb46/sp42 | Is salt available for pupils when they eat hot meals? | No; Yes, but the amount is supervised by an adult = 1  Yes, they serve themselves; Don’t know = 0  Not relevant is not counted | Score 12:  0 or 1 point |
| Sugary and high-fat baked and other goods should be limited to special occasions | Sb49/sp45 | Are birthdays celebrated with cake/ice-cream/sweet buns etc. during the after-school service? | No, birthdays are celebrated in ways other than with food; No, birthdays are not celebrated in the after-school service = 1  Yes, separately for each pupil; Yes, a common celebration weekly; Yes, a common celebration monthly; Don’t know = 0 | Score 13:  0–2 points divided by 2 |
|  | Sb50/sp46 | In the course of the year, how often may pupils eat cake, ice-cream, sweet buns, cookies, etc. during after-school services? | Never; 1 or 2 times/year; 3 or 4 times/year = 1  Once a week or more often; 1–3 times/month; 5–9 times/year; Don’t know = 0 |  |
| Chocolate, confectionery, potato chips and other snacks should not be offered | Sb51/sp47 | In the course of the year, how often may pupils eat chocolate, candy, potato chips, etc. during after-school services? | Never; 1 or 2 times/year; = 1  Once a week or more often; 1–3 times/month; 5–9 times/year; 3 or 4 times/year; Don’t know = 0 | Score 14:  0 or 1 point |
| Eco-friendly practices should be aimed for to achieve minimal food waste and meal options in which plant-based foods and fish are focal | Sb57/sp53 | To what degree is food waste being reduced in the after-school service? | To a large degree; To a fairly large degree = 1  To neither a large nor a small degree; To a fairly low degree; To a low degree; Don’t know = 0  Not relevant is not counted | Score 15:  0–2 points divided by 2 |
|  | Sb58/sp54 | To what degree are environmental concerns taken into account during food service planning? | To a large degree; To a fairly large degree = 1  To neither a large nor a small degree; To a fairly low degree; To a low degree; Don’t know = 0  Not relevant is not counted |  |
| Score for the after-school service |  |  |  | Maximally 15 points |

^a^The Keyhole is a voluntary Nordic label for food.
