# Supplementary material for: Implementation and effectiveness of a school-based intervention to increase adherence to national school meal guidelines: a non-randomised controlled trial
Source: Public Health Nutr. 2024 Jan 2;27(1):e25. doi: 10.1017/S1368980023002938 (PMC10830359; doi:10.1017/S1368980023002938)
Supplement: Randby et al. supplementary material 3 — Randby et al. supplementary material [file S1368980023002938sup003.docx]

**Additional file 3: Scoring of the implementation indices**

# Implementation outcomes measured in the Food Ambassador study

## Index 1 Implementation dimension 1: Quality of delivery

| **Aspect of dimension/construct** | **Data source** | **Question/registry** | **Scoring** | **Variable name in analysis file** |
| --- | --- | --- | --- | --- |
|  |  |  |  |  |
| Session effectiveness (clear and responsive presentations) | Paper-based evaluation of the September trainings (food ambassadors (a) and after-school leaders (SFO leaders)^1^ (s)) | To what degree did the presentations about the project provide a clear understanding of the following:  – Clarity around the aim of the project  – Clarity around the project's relevance for the school  – Clarity about what is expected of you in the project  – Clarity about what the results will be used for  (Response options: Not at all; To a small degree; To some degree; To a large degree) | 1 point for each, if “to a large degree”  Maximum 8 points | d1_a1 to d1_a4  d1_s9 to d1_s12 |
| Session effectiveness (meeting content responsive/sensitive) | Paper-based evaluation of the September trainings (food ambassadors (a) and SFO leaders (s)) | To what degree did you think that the meeting was:  – too theoretical?  – contained too much information?  – only contained already known stuff?  (Response options: Not at all; To a small degree; To some degree; To a large degree) | 1 point for each, if “not at all”  Maximum 6 points | d1_a5 to d1_a7  d1_s13 to d1_s15 |
| Session effectiveness (“sensitive to needs”) | Paper-based evaluation of the September trainings (food ambassadors (a) and SFO leaders (s)) | To what degree did you think that the meeting offered opportunities to share experiences?  (Response options: Not at all; To a small degree; To some degree; To a large degree) | 1 point if “to a large degree”; 0.5 point if “to some degree”  Maximum 2 points | d1_a8_share  d1_s16_share |
| Implementer preparedness and enthusiasm | Paper-based evaluation of the September training sessions (food ambassadors (a) and SFO leaders (s)) | To what degree did you think that the meeting:  – seemed well prepared?  – inspired participation in the project?  (Response options: Not at all; To a small degree; To some degree; To a large degree) | 1 point each if “to a large degree”  Maximum 4 points | d1_a17 to d1_a18  d1_s19 to d1_s20 |
| Implementer enthusiasm | Web-based survey after completion of school workshop (food ambassadors) | How engaged was the project leader about the topics during the September ambassador training?  (Response options: Very unengaged; Unengaged; Neither unengaged nor engaged; Engaged; Very engaged; Not relevant (did not participate)) | 1 point if “engaged or very engaged”  Maximum 1 point | d1_a21 |
| Session effectiveness (generates knowledge) | Web-based survey after completion of school workshop (food ambassadors) | Did you receive sufficient training to conduct the teacher workshop?  (Response options: No; Partly; Yes; Not relevant (did not participate)) | 1 point if “yes”  Maximum 1 point | d1_a22 |
| Session effectiveness (generates knowledge) | Web-based survey after completion of school workshop (food ambassadors or SFO leader) | Did you receive sufficient training to conduct the SFO workshop?  (Response options: No; Partly; Yes; Not relevant (did not participate)) | 1 point if “yes”  Maximum 1 point | d1_as23 |
| Session effectiveness (generates knowledge) | Web-based survey after completion of school workshop  (food ambassadors) | Did you get a good enough overview of the knowledge base for the guideline to be prepared for questions from teacher colleagues?  (Response options: No; Partly; Yes; Not relevant (did not participate)) | 1 point if “yes”  Maximum 1 point | d1_a24 |
| Session effectiveness (generates knowledge) |  | Did you get a good-enough overview of the knowledge base for the guideline to be prepared for questions from SFO colleagues?  (Response options: No; Partly; Yes; Not relevant (did not participate)) | 1 point if “yes”  Maximum 1 point | d1_as25 |
| Sum quality of intervention delivery |  |  | Maximum 25 points | Number of points divided by relevant number of variables |

## Index 2 Implementation dimension 2: Participant responsiveness

| **Aspect of dimension/construct** | **Data source** | **Question/registry** | **Coding** | **Data variable for analysis** |
| --- | --- | --- | --- | --- |
|  |  |  |  |  |
| Participation and involvement (responsiveness to request) | Observation/checklist by principal investigator (PI) | Did the ambassador complete the evaluation form provided in paper at the September training?  (Response options: Yes/No) | 1 point if “yes” | d2_a1 |
| Participation and involvement | Observation/checklist by PI | Response time for download of workshop material (from WeTransfer) by the ambassador  (Response options: Yes, within 2 days; Yes, within a week (before deadline); Yes, but after deadline; never) | 1 point if “yes, within 2 days”; 0.5 point if “yes within a week” | d2_a2 |
| Participation and involvement | Observation/checklist by PI | Did the ambassador send in the action plan after the teacher workshop?  (Response options: Yes, before deadline; Yes, but after deadline; Never) | 1 point if “yes, before deadline”; 0.5 point if “yes, but after deadline” | d2_a3 |
| Participation and involvement (responsiveness to request) | Observation/checklist by PI | Did the SFO leader complete the evaluation form provided in paper at the September training?  (Response options: Yes/No) | 1 point if “yes” | d2_s4 |
| Participation and involvement | Observation/checklist by PI | Did the ambassador (or alternatively the SFO leader) send in the action plan after the SFO workshop?  (Response options: Yes, before deadline; Yes, but after deadline; Never) | 1 point if “yes, before deadline”; 0.5 point if “yes, but after deadline” | d2_s5 |
| Participation | Web-based survey after completion of school workshop (food ambassador) | Did the teachers participate actively during the school workshop?  (Response options: No, only a few; Yes, but fewer than half of them; Yes, more than half of them; Yes, nearly all) | 1 point if “yes, nearly all” | d2_a6_dics |
| Participation | Web-based survey after completion of SFO workshop  (food ambassador/SFO- leader) | Did the SFO staff participate actively during the SFO workshop?  (Response options: No, only a few; Yes, but fewer than half of them; Yes, more than half of them; Yes, nearly all) | 1 point if “yes, nearly all” | d2_s7_disc |
| Engagement | Web-based survey after completion of school workshop (food ambassador) | Was the short film about lunch time allowance from the Directorate of Health shown during the teacher workshop?  (Response options: No; Yes) | 1 point if “yes” | d2_a8_film |
| Engagement | Web-based survey after completion of SFO workshop  (food ambassador/SFO leader) | Was the short film about food safety from the Food Safety Authority shown during the SFO workshop?  (Response options: No; Yes) | 1 point if “yes” | d2_s9_film |
| Participation and involvement (maintaining the interest or attention of participants) | Web-based survey after completion of school workshop  (food ambassador) | Did the workshop format work well as a method to create interest in the topic among teacher colleagues?  (Response options: No; Partly; Yes) | 1 point if “yes” | d2_a10_format |
| Participation and involvement (maintaining the interest or attention of participants) | Web-based survey after completion of SFO workshop (food ambassador/SFO leader) | Did the workshop format work well as a method to create interest in the topic among SFO colleagues?  (Response options: No; Partly; Yes) | 1 point if “yes” | d2_s11_format |
| Engagement | Post-intervention survey (school principals) | Has the school leadership been informed of the brochure about packed food developed in the study?  (Response options: No; Yes; Yes, and it has already been distributed to parents) | 1 point if “yes, and it has already been distributed to parents”; 0.5 point if “yes” | d2_p12_brochure |
| Engagement | Post-intervention survey (school principals) | Has any parent-directed activity related to meals been conducted in connection with the Food Ambassador study?  (Response options: No; No, but we would like to do it in 2020; No, but we have plans to do it in 2020; Yes) | 1 point if “yes”; 0.5 point if “no, but we have plans to do it in 2020” | d2_p13_parents |
| Engagement | Post-intervention survey (school principals) | Has the school conducted any activity for the pupils related to meals in connection with the Food Ambassador study?  (Response options: No; No, but we would like to do it in 2020; No, but we have plans to do it in 2020; Yes) | 1 point if “yes”; 0.5 point if “no, but we have plans to do it in 2020” | d2_p14_pupils |
| Engagement | Post-intervention survey (school principals) | How engaged was the food ambassador in the project period?  (Response options: Very unengaged; Unengaged; Neither unengaged nor engaged; Engaged; Very engaged; Don’t know) | 1 point if “very engaged” or “engaged” | d2_p15_amb_eng |
| Engagement | Post-intervention survey (school principals) | Has participation in the study entailed any changes in routines or practice at school in the last 6 months?  (Response options: No, the school has not been able to prioritize this work in the last 6 months; No, the school already had consistently good practice; Yes, the school has done some changes/adjustments to get even better practice; Yes, the school has done several changes/adjustments to get even better practice; Don’t know) | 1 point if “yes, the school has made several changes/adjustments to get even better practice”; 0.5 point if “yes, the school has made some changes/adjustments to get even better practice” | d2_p16_change_school |
| Engagement | Post-intervention survey (school principals) | Has participation in the study entailed any changes in routines or practice at SFO in the last 6 months?  (Response options: No, SFO has not been able to prioritize this work in the last 6 months; No, SFO already had consistently good practice; Yes, SFO has done some changes/adjustments to get even better practice; Yes, SFO has done several changes/adjustments to get even better practice; Don’t know) | 1 point if “yes, the SFO has done several changes/adjustments to get even better practice”; 0.5 point if “yes, the SFO has done some changes/adjustments to get even better practice” | d2_p17_change_sfo |
| Engagement | Post-intervention survey (school principals) | Are the national guidelines anchored in any of the school’s policy documents?  (Response options: No; No, but we plan to do it in 2020; Yes, new or strengthened anchoring was done in connection with the Food ambassador study; Yes, it has been anchored at our school for some/several years already; Don’t know) | 1 point if “new or strengthened anchoring was done in connection with the Food ambassador study” | d2_p18_guideline |
| Participation and involvement | Post-intervention survey (SFO leaders) | Did the SFO leader participate in the SFO workshop?  (Response options: No; Yes; There was no workshop) | 1 point if “yes” | d2_s19_sfo_lead |
| Engagement | Post-intervention survey (SFO leaders) | Has SFO conducted any activity for the pupils related to meals in connection with the Food Ambassador study?  (Response options: No; No, but we would like to do it in 2020; No, but we have plans to do it in 2020; Yes) | 1 point if “yes”; 0.5 point if “no, but we have plans to do it in 2020” | d2_s20_pupil |
| Engagement | Post-intervention survey (SFO leaders) | Has participation in the study entailed any changes in routines or practice at SFO in the last 6 months?  (Response options: No, SFO has not been able to prioritize this work in the last 6 months; No, SFO already had consistently good practice; Yes, SFO has done some changes/adjustments to get even better practice; Yes, SFO has done several changes/adjustments to get even better practice; Don’t know) | 1 point if “yes, the SFO has done several changes/adjustments to get even better practice”; 0.5 points if “yes, the SFO has done some changes/adjustments to get even better practice” | d2_s21_change_sfo |
| Engagement | Post-intervention survey (SFO leaders) | Is the national guideline anchored in any of SFO’s policy documents?  (Response options: No; No, but we plan to do it in 2020; Yes, new or strengthened anchoring was done in connection with the Food Ambassador study; Yes, it has been anchored at our school for some/several years already; Don’t know) | 1 point if “new or strengthened anchoring was done in connection with the Food Ambassador study” | D2_s22_achor |
| Sum participant responsiveness |  |  | Maximum 22 points |  |

## Index 3 Implementation dimension 3: Dosage

| **Aspect of dimension/construct** | **Data source** | **Question/registry** | **Coding** | **Data variable for analysis** |
| --- | --- | --- | --- | --- |
|  |  |  |  |  |
| Dose received, principal | Observation/checklist by PI | Principal attendance at information meeting (June) | 1 point if “yes” | d3_a1 |
| Dose received, SFO leader | Observation/checklist by PI | SFO leader attendance at  – information meeting (June)  – SFO training (September) | 1 point for each “yes”; but for the SFO training 0.5 point if a step-in attended instead of the SFO leader  Maximum 2 points | d3_s2  d3_s3 |
| Dose received  Food ambassador | Observation/checklist by PI | Food ambassador attendance at  – information meeting (June)  – first ambassador training (September)  – second ambassador training (November) | 1 point for each “yes”;  : 0.5 point if a step-in attended  Maximum 3 points | d3_a4  d3_a5  d3_a6 |
| Dose received, determined by no. of teachers reached in workshop | Web-based survey after completion of school workshop (food ambassadors) | Proportion of class teachers attending the school workshop, as calculated by the following two questions in the questionnaire:  How many class teachers are there at your school?  How many class teachers participated in the workshop? | 1 point if ≥80% of the class teachers participated | d3_a7_part_score |
| Dose received, determined by nr of SFO staff reached  in workshop | Web-based survey after completion of school workshop (food ambassador or SFO leader) | Proportion of SFO staff attending the SFO workshop, as calculated by the following two questions in the questionnaire:  How many SFO staff are there at your school?  How many SFO staff participated in the workshop? | 1 point if ≥80% of the SFO staff participated | d3_s8_part_score |
| Dose received, determined by scope of attendees | Web-based survey after completion of school workshop  (food ambassadors) | Participation by additional employee groups during the school workshop?  (Response options: Yes; No) | 1 point if “yes” | d3_a9_add |
| Sum dosage |  |  | Maximum 9 points |  |

## Index 4 Implementation dimension 4: Fidelity

| **Aspect of dimension/construct** | **Data source** | **Question/registry** | **Coding** | **Data variable for analysis** |
| --- | --- | --- | --- | --- |
|  |  |  |  |  |
| School-level fidelity | Web-based survey after completion of school workshop  (food ambassador) | When was the school workshop conducted?  (Response options: Never; After the November training; After deadline but before November training; Before the deadline 1 November) | 1 point if before the deadline 1 November | d4_a1_date |
| School-level fidelity | Web-based survey after completion of SFO workshop (food ambassador/SFO leader) | When was the SFO workshop conducted?  (Response options: Never; After the November training; after deadline but before November training; Before the deadline 1 November) | 1 point if before the deadline 1 November | d4_s2_date |
| Ambassador fidelity | Web-based survey after completion of school workshop (food ambassador) | Had the person who led the school workshop participated in the September ambassador training?  (Response options: No; Yes) | 1 point if “yes” | d4_a3_train |
| Ambassador fidelity | Web-based survey after completion of SFO workshop (food ambassador/SFO leader) | Had the person who led the SFO workshop participated in one of the September trainings?  (Response options: No; Yes) | 1 point if “yes” | d4_s4_train_corr |
| School-level fidelity | Web-based survey after completion of school workshop (food ambassador) | Were separate workshops conducted for meals during school hours and during SFO, respectively?  (Response options: Yes; No, they were combined; Workshop only for school hours; Workshop only for SFO)  To assess scoring, check comment in follow-up question about why the workshops were combined (e.g. small school OK; no reason provided is not OK) | 1 point if yes, and if a merger is well reasoned | d4_a5_separate |
| School-level fidelity | Web-based survey after completion of school workshop  (food ambassadors) | How much time was set aside for the teacher workshop (approximately)?  (Response options: ≤45 min; 60 min; 1.25 hours; 1.5 hours; 1.75 hours; 2 hours; >2 hours) | 1 point if ≥1.5 hours | d4_a6_time |
| School-level fidelity | Web-based survey after completion of SFO workshop (food ambassador/SFO leader) | How much time was set aside for the SFO workshop (approximately)?  (Response options: ≤45 min; 60 min; 1.25 hours; 1.5 hours; 1h.75 hours; 2 hours; >2 hours) | 1 point if ≥1.5 hours | d4_s7_time |
| Ambassador fidelity | Web-based survey after completion of school workshop (food ambassadors) | Was an introduction to the school workshop given?  (Response options: No; Yes, without use of provided PowerPoint (ppt); Yes, with use of provided ppt; Yes, with use of a ppt adjusted to own needs) | 1 point if “yes, with use of provided ppt” or “yes, with use of a ppt adjusted to own needs” | d4_a8_intro |
| Ambassador fidelity | Web-based survey after completion of SFO workshop (food ambassador/SFO leader) | Was an introduction to the SFO workshop given?  (Response options: No; Yes, without use of provided ppt; Yes, with use of provided ppt; Yes, with use of a ppt adjusted to own needs) | 1 point if “yes, with use of provided ppt” or “yes, with use of a ppt adjusted to own needs” | d4_s9_intro |
| Ambassador fidelity | Web-based survey after completion of school workshop  (food ambassador) | Did each workshop participant fill in the checklist early on in the workshop?  (Response options: No; Yes) | 1 point if “yes” | d4_a10_checkl |
| Ambassador fidelity | Web-based survey after completion of SFO workshop (food ambassador/SFO leader) | Did each workshop participant fill in the checklist early on in the workshop?  (Response options: No; Yes) | 1 point if “yes” | d4_s11_checkl |
| Ambassador fidelity | Web-based survey after completion of school workshop (food ambassadors) | How many of the reflective questions in the guide were used during the workshop?  (Response options: None, or nearly none; Fewer than half; about half; More than half; All, or nearly all) | 1 point if “all or nearly all”; 0.5 point if “more than half” | d4_a12_refl |
| Ambassador fidelity | Web-based survey after completion of SFO workshop (food ambassador/SFO leader) | How many of the reflective questions in the guide were used during the workshop?  (Response options: None, or nearly none; Fewer than half; About half; More than half; All, or nearly all) | 1 point if “all or nearly all”; 0.5 point if “more than half” | d4_s13_refl |
| Ambassador fidelity | Web-based survey after completion of school workshop (food ambassador) | Was there enough time to sum up the areas that you would like to follow up (during school workshop)?  (Response options: No; Partly; Yes; Not relevant/nothing to follow-up) | 1 point if “yes” | d4_a14_sum |
| Ambassador fidelity | Web-based survey after completion of SFO workshop (food ambassador/SFO leader) | Was there enough time to sum up the areas that you would like to follow up (during SFO workshop)?  (Response options: No; Partly; Yes; Not relevant/nothing to follow-up | 1 point if “yes” | d4_s15_sum |
| School-level fidelity | Web-based survey after completion of school workshop (food ambassador) | Were the room/facilities you used for the workshop suitable for the purpose?  (Response options: No; Partly; Yes; Not relevant) | 1 point if “yes” | d4_a16_fac |
| School-level fidelity | Web-based survey after completion of SFO workshop (food ambassador/SFO leader) | Were the room/facilities you used for the workshop suitable for the purpose?  (Response options: No; Partly; Yes; Not relevant) | 1 point if “yes” | d4_s17_fac |
| School-level fidelity | Web-based survey after completion of school workshop (food ambassadors) | Did you get support from the principal to gather class teachers for the workshop?  (Response option: No; Partly; Yes; Not relevant) | 1 point if “yes” | d4_a18_supp |
| School-level fidelity | Web-based survey after completion of school workshop (food ambassadors) | Did you get support from the principal to gather SFO staff for the workshop?  (Response option: No; Partly; Yes; Not relevant) | 1 point if “yes” | d4_s19_supp |
| Ambassador fidelity | Post-intervention survey (school principal) | Has the food ambassador conducted a meeting with the principal about the action plan following the school workshop?  (Response options: No; No, but a meeting is scheduled; Yes) | 1 point if “yes” | d4_p20_sch_meet |
| Ambassador fidelity | Post-intervention survey (school principal) | Has the food ambassador or SFO leader conducted a meeting with the principal about the action plan after the SFO workshop?  (Response options: No; No, but a meeting is scheduled; Yes) | 1 point if “yes” | d4_p21_sfo_meet |
| School-level fidelity | Post-intervention survey (school principal) | Has follow-up of the workshop been a topic in a teachers’ meeting after the workshop?  (Response options: No; No, but we would like to follow it up; No, but a meeting is scheduled; Yes, held in 2019; Yes, held in 2020) | 1 point if “yes, held in 2019” or “yes, held in 2020” | d4_p22_follup_sch |
| School-level fidelity | Post-intervention survey (SFO leader) | Has follow-up of the workshop been a topic in an SFO staff meeting after the workshop?  (Response options: No; No, but we would like to follow it up; No, but a meeting is scheduled; Yes, held in 2019; Yes, held in 2020) | 1 point if “yes, held in 2019” or “yes, held in 2020” | d4_s23_follup_sfo |
| Sum fidelity |  |  | Maximum 23 points |  |

^1^ The Norwegian abbreviation for the after-school service (“SFO”) is used throughout the document, thus the use of “SFO leader” etc.
