# Supplementary figures and images for: Implementation and effectiveness of a school-based intervention to increase adherence to national school meal guidelines: a non-randomised controlled trial
Source: Public Health Nutr. 2024 Jan 2;27(1):e25. doi: 10.1017/S1368980023002938 (PMC10830359; doi:10.1017/S1368980023002938)

Additional file 4. Logic model for the Food Ambassador study

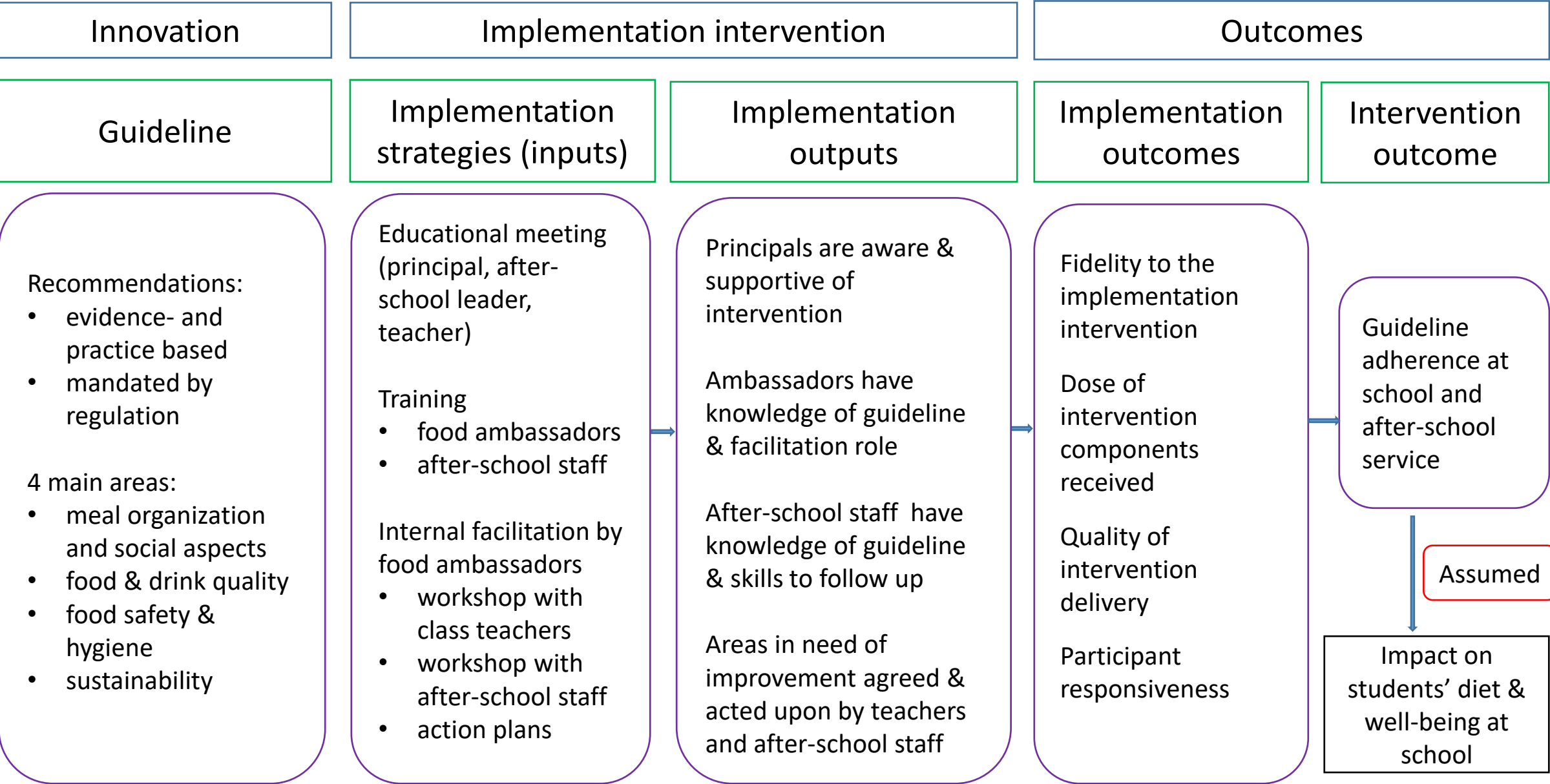

Supplement: Randby et al. supplementary material 4 — Randby et al. supplementary material [file S1368980023002938sup004.pdf]
