# Supplementary material for: Implementation and effectiveness of a school-based intervention to increase adherence to national school meal guidelines: a non-randomised controlled trial
Source: Public Health Nutr. 2024 Jan 2;27(1):e25. doi: 10.1017/S1368980023002938 (PMC10830359; doi:10.1017/S1368980023002938)
Supplement: Randby et al. supplementary material 5 — Randby et al. supplementary material [file S1368980023002938sup005.docx]

**Additional file 5 Supplementary results from data analyses that are not shown in the article**

*School and sample characteristics*

1. Logistic regression was performed to assess whether school socioeconomic profile (SES) was a significant predictor of attrition. SES data were available for 60 of the 67 schools in the baseline sample. For one of the schools that dropped out, SES data were not available.

Dependent variable: drop-out

Independent variable: SES (low vs. high)

SES did not predict drop-out in the sample, χ^2^ (1, *N* = 60) = 1.50, *P* = 0·22.

1. The two-sample Student’s *t*-test was used to test whether there was a significant difference in adherence scores between the two study groups at baseline for the whole baseline sample (*n* = 67).

Results showed that there was no significant difference in baseline adherence scores between the intervention (mean = 0.66, s.d. = 0.08) and comparison group (mean = 0.68, s.d. = 0.08), *t* (65) = −1.08, *P* = 0·28 in the whole baseline sample.

1. Spearman’s rank-order correlation analysis was used to test whether SES was correlated with baseline adherence, in both the whole baseline sample and for the complete cases.

In the whole baseline sample (*n* = 67) SES data were not available for three intervention and four comparison schools due to small school size. Thus, *n* = 60 for this analysis, with *n* = 30 in each group. The results showed that there was no correlation between SES and baseline adherence (*r_s_* = −.04) in the whole sample.

In the complete case sample (*n* = 59) SES data were not available for three intervention and three comparison schools due to small school size. Thus, *n* = 53 for this analysis, with *n* = 28 in the intervention group and *n* = 25 in the comparison group. The results showed that there was no correlation between SES and baseline adherence (*r_s_* = −.12) in the complete case sample.

*Intervention effectiveness*

Sensitivity analysis

To assess the potential impact of selection bias linked with loss to follow-up, we conducted a sensitivity analysis whereby baseline adherence scores were imputed for follow-up scores among the schools that dropped out, thus *n* = 67.

Dependent variable: follow-up adherence score

Independent variables: baseline adherence, study group, interaction term (based on centered variables of baseline adherence and study group)

The results showed the same difference of 4% between the groups (*B* = 0.04, s.e. *B* = 0.01, *t* = 3.5, *P* = 0·001), with *F*[3,63] = 21.03, *P* <0·001.

*Implementation outcomes*

Table s1: Bivariate Correlations^a^ Between Six Implementation Aspects and Change Score

| **Change score and implemementation dimensions/aspects** | **1** | **2** | **3** | **4** | **5** | **6** | **7** |
| --- | --- | --- | --- | --- | --- | --- | --- |
| 1. Change score | − |  |  |  |  |  |  |
| 2. Quality of Delivery | .20 | − |  |  |  |  |  |
| 3. Participation and involvement | .25 | .21 | − |  |  |  |  |
| 4. Engagement | −.07 | .35 | .17 | − |  |  |  |
| 5. Dosage | −.24 | .06 | .09 | −.01 | − |  |  |
| 6. School-level fidelity | .48** | .22 | .53** | −.03 | .07 | − |  |
| 7. Ambassador fidelity | .21 | .35 | .26 | .40* | −.16 | .31 | − |

^a^Pearson’s bivariate correlation is reported for correlations between two variables with normally distributed variables and Spearman’s rank-order correlation is reported for correlations in which at least one variable in non-normally distributed.

** Correlation is significant at the 0·01 level (2-tailed).

* Correlation is significant at the 0·05 level (2-tailed).
