# Supplementary material for: Implementation and effectiveness of a school-based intervention to increase adherence to national school meal guidelines: a non-randomised controlled trial
Source: Public Health Nutr. 2024 Jan 2;27(1):e25. doi: 10.1017/S1368980023002938 (PMC10830359; doi:10.1017/S1368980023002938)

**Additional file 6 Graph of the relationship between change scores and baseline adherence**

**Figure s1.** Relationship between adherence change scores and baseline adherence values in the two study groups in the Food Ambassador Study (*n* = 59)


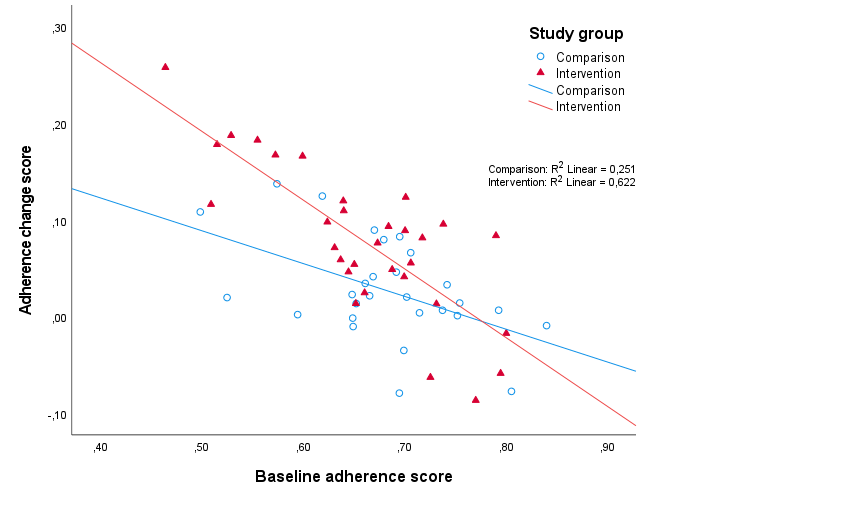

Supplement: Randby et al. supplementary material 6 — Randby et al. supplementary material [file S1368980023002938sup006.docx]
