# Supplementary material for: Implementation and effectiveness of a school-based intervention to increase adherence to national school meal guidelines: a non-randomised controlled trial
Source: Public Health Nutr. 2024 Jan 2;27(1):e25. doi: 10.1017/S1368980023002938 (PMC10830359; doi:10.1017/S1368980023002938)
Supplement: Randby et al. supplementary material 7 — Randby et al. supplementary material [file S1368980023002938sup007.docx]

**Additional file 7: Adherence change for each guideline domain**

Average adherence score for each guideline recommendation/domain at baseline and follow-up in the intervention and comparison groups collected from the principal and after-school leader questionnaires

1. Principal questionnaire results – intervention group

|  |  | **Baseline** | | **Follow-up** | | **Difference** | |
| --- | --- | --- | --- | --- | --- | --- | --- |
| **Recommendations** | ***n*** | **Mean** | **s.d.** | **Mean** | **s.d.** | **Mean change** | **s.d.** |
| Meals should be arranged so as to be conducted at 3- to 4-hourly intervals | 31 | 0.89 | 0.21 | 0.87 | 0.26 | −0.02 | 0.25 |
| Physical arrangements should be made for meals that promote enjoyment of meals, socialization, well-being and health | 31 | 0.47 | 0.18 | 0.51 | 0.21 | 0.03 | 0.20 |
| Pupils should be given enough time to eat – at least 20 minutes | 31 | 0.53 | 0.45 | 0.71 | 0.40 | 0.18 | 0.36 |
| Pupils should be supervised by an adult at mealtimes | 31 | 0.98 | 0.09 | 0.98 | 0.09 | 0.00 | 0.00 |
| Cold drinking water should be available at all times as a thirst quencher and to accompany meals | 31 | 0.61 | 0.21 | 0.74 | 0.25 | 0.13 | 0.21 |
| Pupils should be offered schemes that ensure daily access to vegetables, fruit or berries | 31 | 0.52 | 0.51 | 0.61 | 0.50 | 0.10 | 0.27 |
| Pupils should be offered schemes that ensure access to milk to accompany meals: reduced-fat semi-skimmed milk (0.7% fat), semi-skimmed milk (1% fat) and/or skimmed milk (0.1% fat) | 31 | 0.74 | 0.25 | 0.79 | 0.25 | 0.05 | 0.25 |
| Arrangements should be made to ensure hand-washing before meals | 31 | 0.48 | 0.31 | 0.59 | 0.33 | 0.12 | 0.29 |
| The storage, preparation, serving and labeling of food must be carried out in compliance with rules and recommendations issued by the Norwegian Food Safety Authority | 31 | 0.61 | 0.33 | 0.68 | 0.33 | 0.06 | 0.27 |
| Carbonated soft drinks, squash and other beverages containing added sugar or artificial sweeteners and caffeinated beverages should not be offered | 31 | 0.55 | 0.51 | 0.52 | 0.51 | −0.03 | 0.42 |
| Sugary and high-fat baked and other goods should be limited to special occasions | 31 | 0.68 | 0.28 | 0.73 | 0.28 | 0.05 | 0.26 |
| Chocolate, confectionery, potato chips and other snacks should not be offered | 31 | 0.74 | 0.44 | 0.87 | 0.34 | 0.13 | 0.43 |

|  |
| --- |
|  |
|  |
|  |
| 1. Principal questionnaire results – comparison group  \|  \|  \| **Baseline** \| \| **Follow-up** \| \| **Difference** \| \| \| --- \| --- \| --- \| --- \| --- \| --- \| --- \| --- \| \| **Recommendations** \| ***n*** \| **Mean** \| **s.d.** \| **Mean** \| **s.d.** \| **Mean change** \| **s.d.** \| \| Meals should be arranged so as to be conducted at 3- to 4-hourly intervals \| 28 \| 0.75 \| 0.35 \| 0.77 \| 0.32 \| 0.02 \| 0.25 \| \| Physical arrangements should be made for meals that promote enjoyment of meals, socialization, well-being and health \| 28 \| 0.51 \| 0.18 \| 0.45 \| 0.16 \| −0.06 \| 0.20 \| \| Pupils should be given enough time to eat – at least 20 minutes \| 28 \| 0.86 \| 0.27 \| 0.71 \| 0.40 \| −0.14 \| 0.36 \| \| Pupils should be supervised by an adult at mealtimes \| 28 \| 0.98 \| 0.09 \| 0.98 \| 0.09 \| 0.00 \| 0.00 \| \| Cold drinking water should be available at all times as a thirst quencher and to accompany meals \| 28 \| 0.88 \| 0.22 \| 0.77 \| 0.25 \| −0.11 \| 0.21 \| \| Pupils should be offered schemes that ensure daily access to vegetables, fruit or berries \| 28 \| 0.68 \| 0.48 \| 0.68 \| 0.48 \| 0.00 \| 0.27 \| \| Pupils should be offered schemes that ensure access to milk to accompany meals: reduced-fat semi-skimmed milk (0.7% fat), semi-skimmed milk (1% fat) and/or skimmed milk (0.1% fat) \| 28 \| 0.77 \| 0.25 \| 0.75 \| 0.25 \| −0.02 \| 0.25 \| \| Arrangements should be made to ensure hand-washing before meals \| 28 \| 0.44 \| 0.28 \| 0.49 \| 0.29 \| 0.06 \| 0.29 \| \| The storage, preparation, serving and labelling of food must be carried out in compliance with rules and recommendations issued by the Norwegian Food Safety Authority \| 27 \| 0.63 \| 0.36 \| 0.70 \| 0.29 \| 0.07 \| 0.27 \| \| Carbonated soft drinks, squash and other beverages containing added sugar or artificial sweeteners and caffeinated beverages should not be offered \| 28 \| 0.54 \| 0.51 \| 0.64 \| 0.49 \| 0.11 \| 0.42 \| \| Sugary and high-fat baked and other goods should be limited to special occasions \| 28 \| 0.69 \| 0.26 \| 0.71 \| 0.34 \| 0.02 \| 0.26 \| \| Chocolate, confectionery, potato chips and other snacks should not be offered \| 28 \| 0.79 \| 0.42 \| 0.82 \| 0.39 \| 0.04 \| 0.43 \| |
|  |
|  |
|  |
|  |
| 1. After-school leader questionnaire results – intervention group |
|  |
|  |
| \|  \|  \| **Baseline** \| \| **Follow-up** \| \| **Difference** \| \| \| --- \| --- \| --- \| --- \| --- \| --- \| --- \| --- \| \|  \| ***n*** \| **Mean** \| **s.d.** \| **Mean** \| **s.d.** \| **Mean**  **change** \| **s.d.** \| \| Meals should be arranged so as to be conducted at 3- to 4-hourly intervals \| 31 \| 0.71 \| 0.46 \| 0.61 \| 0.50 \| −0.10 \| 0.54 \| \| Cold drinking water should be available at all times as a thirst quencher and to accompany meals \| 31 \| 1.00 \| 0.00 \| 1.00 \| 0.00 \| 0.00 \| 0.00 \| \| Pupils should be offered schemes that ensure daily access to vegetables, fruit or berries \| 31 \| 0.31 \| 0.31 \| 0.35 \| 0.29 \| 0.05 \| 0.20 \| \| Pupils should be offered schemes that ensure access to milk to accompany meals: reduced-fat semi-skimmed milk (0.7% fat), semi-skimmed milk (1% fat) and/or skimmed milk (0.1% fat) \| 31 \| 0.85 \| 0.24 \| 0.88 \| 0.24 \| 0.03 \| 0.25 \| \| The storage, preparation, serving and labelling of food must be carried out in compliance with rules and recommendations issued by the Norwegian Food Safety Authority \| 31 \| 0.39 \| 0.27 \| 0.57 \| 0.28 \| 0.19 \| 0.27 \| \| The needs of pupils with food allergies or food intolerances should be accommodated \| 29 \| 0.90 \| 0.31 \| 0.97 \| 0.19 \| 0.07 \| 0.37 \| \| Carbonated soft drinks, squash and other beverages containing added sugar or artificial sweeteners and caffeinated beverages should not be offered \| 31 \| 0.23 \| 0.43 \| 0.65 \| 0.49 \| 0.42 \| 0.50 \| \| Bread and cereals in school meals should be high in fibre and whole grains and low in fat, sugar and salt \| 31 \| 0.85 \| 0.23 \| 0.90 \| 0.18 \| 0.05 \| 0.15 \| \| Bread toppings/spreads offered to pupils should be varied and always include fish and vegetables \| 31 \| 0.70 \| 0.21 \| 0.81 \| 0.16 \| 0.11 \| 0.17 \| \| Any hot meals served should be a variety of fish, meat and vegetarian dishes \| 31 \| 0.58 \| 0.34 \| 0.74 \| 0.31 \| 0.16 \| 0.35 \| \| Cooking oils and liquid and soft margarine should be used instead of hard margarine and butter \| 31 \| 0.76 \| 0.36 \| 0.79 \| 0.28 \| 0.03 \| 0.31 \| \| Low-salt/sodium foods should be given priority and the use of salt/sodium as seasoning in food preparation and on meals should be limited \| 31 \| 0.97 \| 0.18 \| 1.00 \| 0.00 \| 0.03 \| 0.18 \| \| Sugary and high-fat baked and other goods should be limited to special occasions \| 31 \| 0.58 \| 0.39 \| 0.69 \| 0.40 \| 0.11 \| 0.36 \| \| Chocolate, confectionery, potato chips and other snacks should not be offered \| 31 \| 0.77 \| 0.43 \| 0.90 \| 0.30 \| 0.13 \| 0.43 \| \| Eco-friendly practices should be aimed for to achieve minimal food waste and meal options in which plant-based foods and fish are focal \| 31 \| 0.42 \| 0.39 \| 0.45 \| 0.35 \| 0.03 \| 0.46 \| |
|  |

1. After-school leader questionnaire results – comparison group

|  |  | **Baseline** | | **Follow-up** | | **Difference** | |
| --- | --- | --- | --- | --- | --- | --- | --- |
| **Recommendations** | ***n*** | **Mean** | **s.d.** | **Mean** | **s.d.** | **Mean**  **change** | **s.d.** |
| Meals should be arranged so as to be conducted at 3- to 4-hourly intervals | 28 | 0.82 | 0.39 | 0.79 | 0.42 | −0.04 | 0.51 |
| Cold drinking water should be available at all times as a thirst quencher and to accompany meals | 28 | 1.00 | 0.00 | 1.00 | 0.00 | 0.00 | 0.00 |
| Pupils should be offered schemes that ensure daily access to vegetables, fruit or berries | 28 | 0.32 | 0.37 | 0.30 | 0.34 | −0.02 | 0.35 |
| Pupils should be offered schemes that ensure access to milk to accompany meals: reduced-fat semi-skimmed milk (0.7% fat), semi-skimmed milk (1% fat) and/or skimmed milk (0.1% fat) | 28 | 0.64 | 0.37 | 0.67 | 0.33 | 0.02 | 0.26 |
| The storage, preparation, serving and labelling of food must be carried out in compliance with rules and recommendations issued by the Norwegian Food Safety Authority | 28 | 0.40 | 0.25 | 0.47 | 0.32 | 0.07 | 0.30 |
| The needs of pupils with food allergies or food intolerances should be accommodated | 26 | 0.92 | 0.27 | 0.96 | 0.20 | 0.04 | 0.20 |
| Carbonated soft drinks, squash and other beverages containing added sugar or artificial sweeteners and caffeinated beverages should not be offered | 28 | 0.39 | 0.50 | 0.57 | 0.50 | 0.18 | 0.55 |
| Bread and cereals in school meals should be high in fibre and whole grains and low in fat, sugar and salt | 28 | 0.82 | 0.29 | 0.87 | 0.21 | 0.05 | 0.20 |
| Bread toppings/spreads offered to pupils should be varied and always include fish and vegetables | 24 | 0.67 | 0.21 | 0.61 | 0.26 | −0.06 | 0.16 |
| Any hot meals served should be a variety of fish, meat and vegetarian dishes | 24 | 0.60 | 0.29 | 0.69 | 0.29 | 0.08 | 0.24 |
| Cooking oils and liquid and soft margarine should be used instead of hard margarine and butter | 24 | 0.58 | 0.41 | 0.75 | 0.33 | 0.17 | 0.38 |
| Low-salt/sodium foods should be given priority and the use of salt/sodium as seasoning in food preparation and on meals should be limited | 24 | 1.00 | 0.00 | 1.00 | 0.00 | 0.00 | 0.00 |
| Sugary and high-fat baked and other goods should be limited to special occasions | 28 | 0.59 | 0.41 | 0.68 | 0.41 | 0.09 | 0.31 |
| Chocolate, confectionery, potato chips and other snacks should not be offered | 28 | 0.71 | 0.46 | 0.82 | 0.39 | 0.11 | 0.42 |
| Eco-friendly practices should be aimed for to achieve minimal food waste and meal options in which plant-based foods and fish are focal | 26 | 0.44 | 0.36 | 0.54 | 0.31 | 0.10 | 0.28 |
